# Circadian regulation of mitochondrial uncoupling and lifespan

**DOI:** 10.1038/s41467-020-15617-x

**Published:** 2020-04-21

**Authors:** Matt Ulgherait, Anna Chen, Sophie F. McAllister, Han X. Kim, Rebecca Delventhal, Charlotte R. Wayne, Christian J. Garcia, Yocelyn Recinos, Miles Oliva, Julie C. Canman, Martin Picard, Edward Owusu-Ansah, Mimi Shirasu-Hiza

**Affiliations:** 1 Department of Genetics and Development, Columbia University Vagelos College of Physicians and Surgeons, New York, NY 10032 USA; 2 Columbia College, New York, NY 10027 USA; 3 Department of Neurology, Columbia University Vagelos College of Physicians and Surgeons, New York, NY 10032 USA; 4 Department of Physiology and Cellular Biophysics, Columbia University Vagelos College of Physicians and Surgeons, New York, NY 10032 USA; 5 Department of Systems Biology, Columbia University Vagelos College of Physicians and Surgeons, New York, NY 10032 USA; 6 Department of Pathology and Cell Biology, Columbia University Vagelos College of Physicians and Surgeons, New York, NY 10032 USA; 7 Departments of Psychiatry and Neurology, Columbia University Vagelos College of Physicians and Surgeons, New York, NY 10032 USA

**Keywords:** Circadian rhythms, Ageing, Stem cells, Genetics, Ageing

## Abstract

Because old age is associated with defects in circadian rhythm, loss of circadian regulation is thought to be pathogenic and contribute to mortality. We show instead that loss of specific circadian clock components Period (Per) and Timeless (Tim) in male *Drosophila* significantly extends lifespan. This lifespan extension is not mediated by canonical diet-restriction longevity pathways but is due to altered cellular respiration via increased mitochondrial uncoupling. Lifespan extension of per mutants depends on mitochondrial uncoupling in the intestine. Moreover, upregulated uncoupling protein UCP4C in intestinal stem cells and enteroblasts is sufficient to extend lifespan and preserve proliferative homeostasis in the gut with age. Consistent with inducing a metabolic state that prevents overproliferation, mitochondrial uncoupling drugs also extend lifespan and inhibit intestinal stem cell overproliferation due to aging or even tumorigenesis. These results demonstrate that circadian-regulated intestinal mitochondrial uncoupling controls longevity in *Drosophila* and suggest a new potential anti-aging therapeutic target.

## Abstract (web summary)

Disruption of different components of molecular circadian clocks has varying effects on health and lifespan of model organisms. Here the authors show that loss of *period* extends life in drosophila melanogaster.

## Introduction

**Fig. 1:**
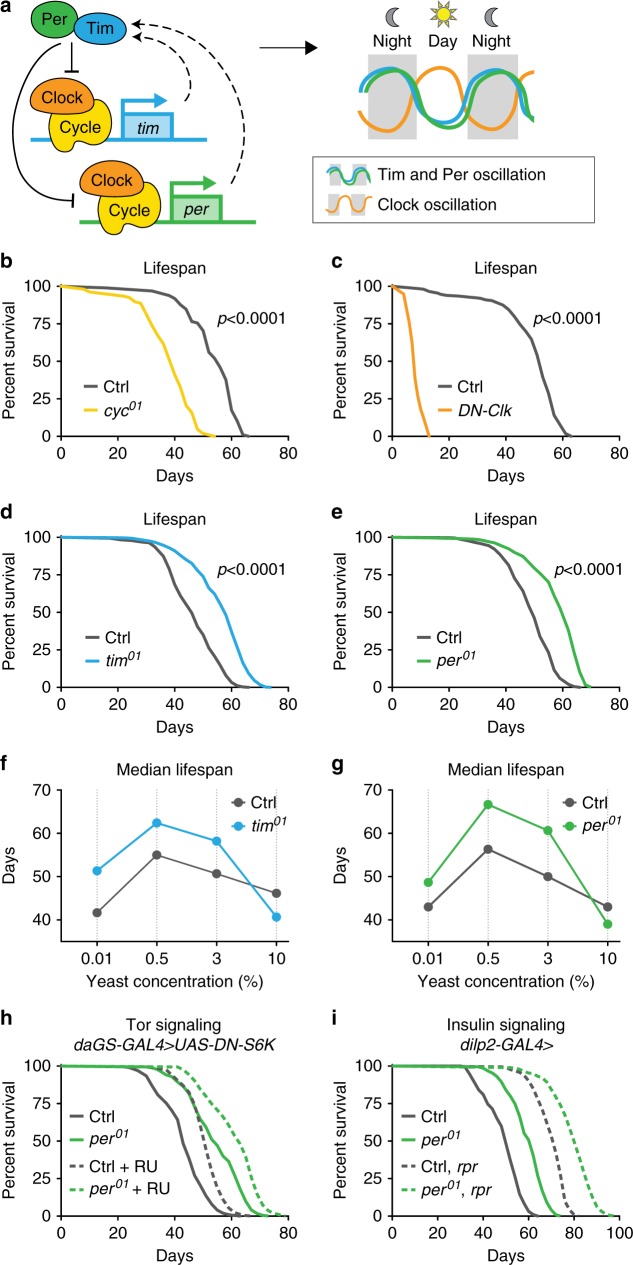
Loss of the repressive arm of the transcriptional circadian clock extends male lifespan. **a** Schematic of core molecular clock components and circadian transcriptional feedback loop. Relative to controls (gray), mutant males lacking *Cyc* function (yellow, **b**) or expressing dominant-negative clock (*daGS* > *UAS-DN-Clock* flies fed RU486, orange, **c**) show reduced lifespan (*p* < 0.0001 for each circadian mutant vs. control). In contrast, *tim*^01^ mutants (blue, **d**) and *per*^01^ mutants (green, **e**) live longer than controls (gray), even on diets with different concentrations of yeast extract (**f**, **g**), except for the highest yeast diet (*p* < 0.001 for each circadian mutant vs. control on each diet). **h** *per*^01^ mutants with *daGS* > *UAS****-****DN****-****S6K* fed either RU486 (dashed lines) or vehicle (solid lines) exhibited similar lifespan extension relative to controls containing *daGS* > *UAS-DN-S6K*. **i** *per*^01^ mutant males containing either the *dilp2-GAL4* driver alone (solid lines) or *dilp2-GAL4* > *UAS-reaper* and ablated for insulin-producing cells (dashed lines) exhibit similar lifespan extension relative to controls. See Supplementary Table 1 for *n* and *p* values for lifespan experiments, particularly multicurve comparisons; *p* values were obtained by log-rank analysis.

Most animals exhibit behavior and physiologies linked to a 24-h circadian rhythm, controlled by endogenous circadian clocks. These clocks are composed of transcriptional feedback loops regulating the expression of genes controlling diverse cellular functions. In *Drosophila*, the circadian transcriptional activators Clock and Cycle induce the oscillating expression of hundreds of target genes, including *period* (*per*) and *timeless* (*tim*), which encode repressors of Clock and Cycle activity (Fig. 1a). Because organisms lose circadian rhythmicity with age, it has been hypothesized that loss of circadian regulation contributes to aging and limits lifespan. Specifically, there has been interest in the impact of circadian-regulated metabolism on lifespan and aging, as many mechanisms regulating organismal lifespan involve large metabolic changes, including circadian-regulated metabolic genes^1,2^. Most reports investigating core molecular clock components and lifespan examined the circadian transcriptional activators (i.e., Clock or Cycle) and found that disruption of these activators led to metabolic dysfunction and shortened lifespan^3–7^. Studies examining loss of circadian transcriptional repressors (i.e. Period, Timeless, or Cryptochrome) and the impact on metabolism, healthspan, and lifespan have been more controversial^5,8–12^. While a number of studies identified the detrimental effects of loss of the transcriptional repressor *per2* in the mouse^13,14^ or *per* in *Drosophila*^9,15,16^, loss of *per2* may also have beneficial effects on specific aspects of healthspan and lifespan^12,17–19^. For example, loss of *per2* in the whole mouse has been associated with increased metabolic rate, lowered fat storage, increased leptin levels, and decreased insulin resistance compared with control animals, indicating a possibly favorable metabolic state under ad libitum feeding conditions^17,20^. Thus, while loss of circadian regulation has pathological effects, loss of circadian regulation in specific genetic and environmental contexts may have metabolic advantages. The specific mechanisms underlying circadian-regulated metabolism and their roles in aging and longevity remain unclear.

Here, we show that loss of the repressive arm of the core circadian clock extends male *Drosophila* lifespan. Loss of *period* also induces a highly active metabolic state characterized by increased mitochondrial uncoupling; this lifespan extension is due to upregulation of the endogenous mitochondrial uncoupling protein (UCP) UCP4C, specifically in the intestine. Loss of *per* or upregulation of UCP4C attenuates age-related decline in gut homeostasis. These genetic phenotypes, including longevity extension and improved gut homeostasis, are recapitulated by feeding *Drosophila* low doses of mitochondrial uncoupling drugs.

## Results

### Loss of *tim* or *per* extends lifespan in male *Drosophila*

To investigate how loss of specific circadian regulators influence aging, we examined the lifespans of four established arrhythmic *Drosophila* mutants (Fig. 1a): three genomic mutants, *cycle* (*cyc*^01^), *period* (*per*^01^), and *timeless* (*tim*^01^), and flies ubiquitously expressing a dominant-negative form of *Clock* (*DN-Clock*)^21^. Consistent with other reports^3–6^, functional disruption of the circadian transcriptional activators Cycle or Clock shortened the lifespan of male flies relative to controls (Fig. 1b, c). In contrast, functional disruption of the circadian transcriptional repressors Per and Tim significantly increased lifespan (15–20%) relative to controls; this appeared to be a male-specific effect (Fig. 1d, e). To confirm that lifespan extension was due to the loss of Per protein, we restored Per expression using the *UAS*–*GAL4* system^22^. Expressing either of the two independent *period* transgenes using either the *timeless*–*GAL4* driver or the *ubiquitin*–*GAL4* driver in the *per*^01^ null background reverted *per* mutant lifespan to that of control animals (Supplementary Fig. 1A, B). Thus, loss of Per expression extends lifespan.

**Fig. 2:**
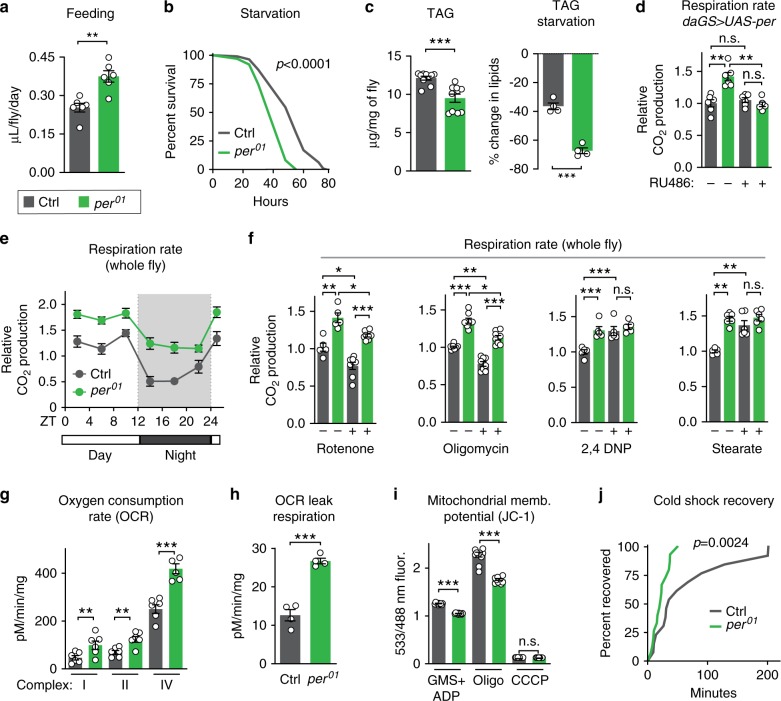
*period* mutants exhibit high metabolic rate due to mitochondrial uncoupling. Relative to controls (gray), *per*^01^ mutants (green) exhibited: **a** increased feeding rate (*n* = 6 vials of ten flies/condition, *p* < 0.01); **b** decreased survival upon starvation (*n* ≥ 99 flies per condition, *p* < 0.001); **c** lower baseline levels of lipids (left) and increased rate of lipid utilization after 24 h of starvation (right), as shown by quantification of triacylglyceride (TAG) levels (*n* ≥ 4 samples/condition, 5 flies/sample, both *p* < 0.0001); **d** increased respiration, which was reverted by ubiquitous overexpression of Per during adulthood (*n* = 6 groups of 10 flies per condition); **e** higher CO_2_ production over the circadian day (*n* = 6 groups of 10 flies/condition and timepoint); **f** higher respiration rates after 24 h of feeding with rotenone and oligomycin, but not with 2,4-DNP, or stearic acid (*n* ≥ 5 groups of 10 flies/condition); **g** increased oxygen consumption rate when stimulated through complexes I, II, and IV relative to controls (*n* = 4–6 oxygraph runs per condition); **h** increased leak respiration, using high-resolution respirometry on purified mitochondria (*n* = 5 oxygraph runs per condition, *p* < 0.001); **i** lower membrane potential, measured by JC-1 staining of purified mitochondria (*n* = 10 mitochondrial preps per condition, *p* < 0.001); and **j** faster recovery from cold shock (*n* = 26–30 flies per condition, *p* < 0.001). See Supplementary Information for *n* if not listed here; ^n.s.^*p* > 0.05, *p < 0.05, **p < 0.01, ***p < 0.001; *p* values were obtained by unpaired two-tailed *t*-test (**a**, **c**, **e**, **g**–**i**), ANOVA followed by Tukey’s post-hoc test (**d**, **f**), and log-rank analysis (**b**, **j**); error bars represent SEM.

The classic method of lifespan extension is dietary restriction (DR). We showed previously that *per*^01^ and *tim*^01^ mutants are not diet-restricted—that is, these mutants eat more, not less, than controls (ref. ^19^; see also Fig. 2a). However, loss of Per and Tim might mimic physiological changes associated with DR. If so, DR should not further extend the lifespan of male *per*^01^ and *tim*^01^ mutants. In *Drosophila*, DR-mediated lifespan extension is accomplished by titration of protein (yeast extract (YE)). To test response to DR, we fed *per*^01^ and *tim*^01^ null mutants and controls four different concentrations of YE: 0.01% (low), 0.5% (DR), 3% (standard), and 10% (high). As we showed previously for female *per*^01^ and *tim*^01^ mutants^10^, male *per*^01^ and *tim*^01^ mutants exhibited DR-induced lifespan extensions similar to controls and lived longer than controls on most dietary protein concentrations (Fig. 1f, g). The lifespan of these circadian mutants was similar to that of control animals only at very high yeast concentrations (10%), which shortens lifespan. Thus, the extended longevity of *per*^01^ mutants appears to be independent of DR.

We next tested if *per*^01^ mutants exhibit canonical changes in DR-associated mechanisms of longevity, including: decreased insulin signaling, measured by decreased phosphorylation of Akt protein; decreased TOR signaling, measured by decreased phosphorylation of S6K; and increased autophagy, measured by increased lipidation of Atg8. Surprisingly, *per*^01^ males were atypical for these hallmarks of longevity and, at some points in the circadian cycle, exhibited the opposite phenotype as predicted for long-lived mutants (Supplementary Fig. 1C). We further used genetic manipulations to determine if the inhibition of TOR signaling or the inhibition of insulin-like signaling (ILS) is responsible for the longevity of *per*^01^ mutants. To test the inhibition of TOR signaling, we ubiquitously suppressed TORC1 signaling in adulthood by inducible (RU486-mediated) overexpression of a dominant-negative form of the downstream kinase S6K, which is known to extend lifespan^23^. The inhibition of TORC1 signaling extended the lifespans of both control animals and *per*^01^ mutants to a similar magnitude (Fig. 1h), suggesting that the longevity of *per*^01^ mutants is independent of TORC1 inhibition. Feeding RU486 to either controls or *per*^01^ mutants lacking the UAS transgene had no influence on lifespan (Supplementary Fig. 1D). To test the inhibition of ILS, we performed partial genetic ablation of insulin-producing cells in the fly brain using the proapoptotic gene *reaper*^24^. This extended the lifespans of both control animals and *per*^01^ mutants to a similar magnitude (Fig. 1i), suggesting that *per*^01^-associated lifespan extension is independent of insulin-like signaling inhibition. Together, these results suggest that the longevity phenotype of *per*^01^ males is not due to canonical longevity mechanisms but due to a different, independent pathway.

### *per* mutants exhibit a high metabolic rate

As metabolism and lifespan are linked, we set out to investigate the metabolism of long-lived *per*^01^ mutants. As shown in our previous work^19^, *per*^01^ males exhibited hyperphagia (increased feeding), decreased starvation resistance, low levels of lipid storage, and increased starvation-induced lipid utilization relative to controls (Fig. 2a–c, Supplementary Fig. 2A). Because *per*^01^ mutants eat more but are leaner than controls, we tested for hyperactive metabolic rate by measuring CO_2_ production. Consistent with our previous characterization, *per*^01^ mutants produced more CO_2_ throughout the circadian cycle relative to controls, which was reverted by exogenous Per expression (Fig. 2d, e); respiration rate was not affected in RU486 feeding controls (Supplementary Fig. 2B). Respiration rate is significantly affected by the mitochondrial function of oxidative phosphorylation. To determine if increased oxidative phosphorylation caused this increased metabolic output, flies were fed sublethal doses of mitochondrial complex inhibitors: rotenone, which blocks complex I of the mitochondrial electron transport chain (ETC), or oligomycin, which blocks complex V, the F_0_F_1_ ATP synthase. While these compounds inhibited the CO_2_ output of both control and *per*^*01*^ animals to a similar degree, *per*^01^ flies still had higher respiration rates than controls (Fig. 2f). This suggests that their increased respiration is independent of mitochondrial ATP synthesis and instead implicates a different mitochondrial function, such as mitochondrial uncoupling.

Mitochondrial uncoupling increases respiration by dissipation of the proton gradient and uncoupling of oxidative phosphorylation from ATP synthesis, creating futile cycles of respiration and generating heat, as in mammalian brown fat. To test the effects of mitochondrial uncoupling on respiration, we fed *per*^01^ mutants and control flies two different mitochondrial uncoupling compounds: 2,4-dinitrophenol (2,4-DNP), a proton ionophore that dissipates the proton gradient; and stearic acid, which induces mitochondrial uncoupling by activating endogenous UCP activity. Treatment with either drug increased the respiration of control flies to levels similar to those of *per*^01^ mutants but did not increase the respiration of *per*^01^ mutants (Fig. 2f). These results suggest that the increased respiration of *per*^01^ mutants is due to increased mitochondrial uncoupling and that *per*^01^ mutants may already be maximally uncoupled within viable parameters. Higher doses of uncoupling drugs were lethal to both genotypes.

To directly test whether *per*^01^ mutants were mitochondrially uncoupled relative to controls, we assessed mitochondrial function in vitro. We purified mitochondria from *per*^01^ mutants and controls and measured O_2_ consumption. *per*^01^ mutant mitochondria exhibited increased O_2_ consumption when initiated through ETC complexes I, II, and IV (Fig. 2g). This increased O_2_ consumption was not due to increased mitochondrial ETC protein abundance or increased enzymatic activity (Supplementary Fig. 2C, D). Instead, *per*^01^ mitochondria exhibited two critical hallmarks of mitochondrial uncoupling relative to control mitochondria: increased leak respiration, measured by higher oxygen consumption after oligomycin treatment (Fig. 2h); and decreased membrane potential, or disrupted proton gradient, measured by the membrane potential dye sensor JC-1, both during steady-state ATP generation and after inhibition by oligomycin (Fig. 2i). Finally, to assess heat generation, another hallmark of mitochondrial uncoupling, we performed cold shock recovery assays on *per*^01^ mutants and control animals (Fig. 2j). After 1 h of cold shock at 4 °C, *per*^01^ mutants recovered significantly faster than controls, suggesting that *per*^01^ mutants may generate more heat than controls. Thus, loss of Per protein increases mitochondrial uncoupling.

### Uncoupling protein UCP4C is required for *per* mutant longevity

**Fig. 3:**
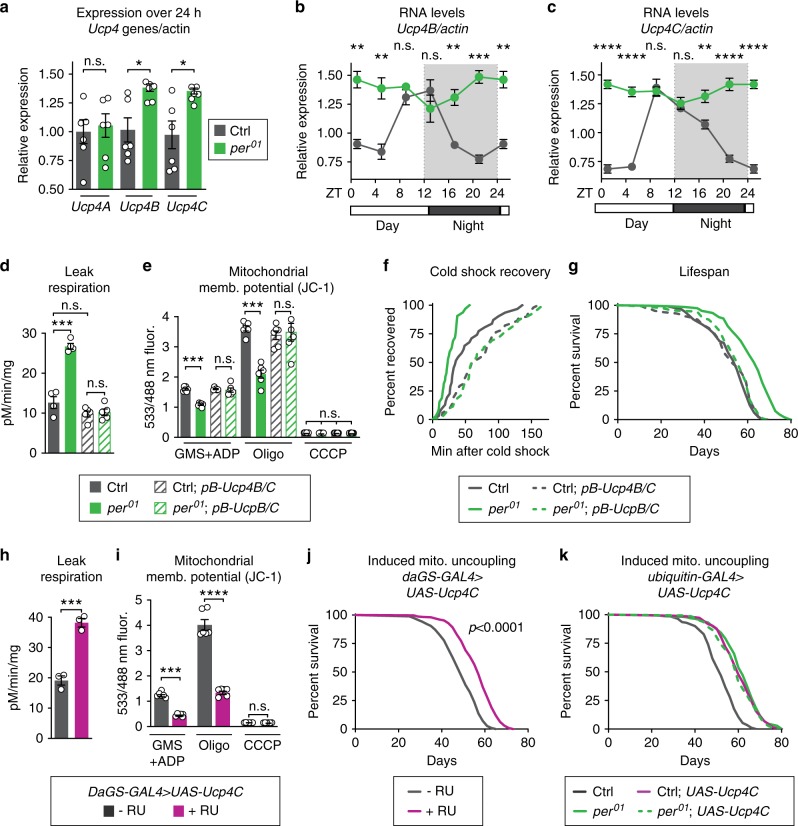
UCP4C is necessary for *period* mutant lifespan and sufficient to extend wild-type lifespan. Relative to controls (gray), *per*^01^ mutants (green) exhibited: **a** higher expression of *Ucp4B* and *Ucp4C* but not *Ucp4A*; and constitutively high expression of **b** *Ucp4B* and **c** *Ucp4C*, both of which are circadian-regulated in controls. Relative to controls (gray), *per*^01^ mutants (green) also exhibited the following phenotypes, which were reverted by suppression of *Ucp4B/C* expression, comparing flies with (dashed lines) or without (solid lines) piggyback mutation of *Ucp4B/C*: **d** increased leak respiration by purified mitochondria; **e** decreased mitochondrial membrane potential; **f** faster cold shock recovery; and **g** increased lifespan. Relative to vehicle-fed controls (gray), *daGS* > *UAS-Ucp4C* flies fed RU486 to induce constitutive UCP4C overexpression (magenta) exhibited: **h** higher leak respiration (*p* < 0.001); **i** lower mitochondrial membrane potential (*p* < 0.001); and **j** increased lifespan (*p* < 0.0001). **k** Ubiquitous overexpression of UCP4C in otherwise wild-type flies was sufficient to extend lifespan (gray, dashed) relative to driver-only controls (gray, solid). In *per*^01^ mutants (green, solid), overexpression of UCP4C did not further extend the lifespan of *per*^01^ mutants (green, dashed). See Supplementary Table 1 for *n* and statistical analysis of lifespans, particularly multicurve comparisons; ^n.s.^*p* > 0.05, *p < 0.05, **p < 0.01, ***p < 0.001, ****p < 0.001; *p* values were obtained by unpaired two-tailed *t*-test (**a**, **h**, **i**), ANOVA followed by Tukey’s post-hoc test (**d**, **e**), and log-rank analysis (**f**, **g**, **j**, **k**); error bars represent SEM.

To determine if this increased mitochondrial uncoupling is required for the lifespan extension of *per*^01^ mutants, we genetically manipulated the expression of endogenous proteins that cause mitochondrial uncoupling. Like many animals, *Drosophila* can undergo mitochondrial uncoupling by induction of UCPs, including UCP4A, B, and C^25,26^. Of these, we found that the expression of *Ucp4B* and *Ucp4C* is circadian-regulated in control flies and constitutively high in *per*^01^ mutants (Fig. 3a–c). To test the role of UCPs in the metabolic phenotype observed in *per*^01^ mutants, we first disrupted the expression of both UCP4B/C proteins using a mutant containing a *piggyBac* transposon in the intergenic region between these two closely linked *Ucp4* genes (Supplementary Fig. 3A, B). Next, to determine if *period* nulls exhibit true mitochondrial uncoupling via classic mitochondrial UCPs, we tested stimulation of mitochondrial respiration in the presence of palmitate and reversal by the UCP inhibitor guanosine nucleotide (GTP) suppression of respiration. Palmitate and GTP are respectively known to stimulate and suppress classic mitochondrial UCPs^27–29^. Indeed, *per*^01^ flies showed significantly increased respiration in the presence of palmitate, which was reversed by the addition of GTP (Supplementary Fig. 3C). *per*^01^ flies with disrupted UCP4B/C expression showed no response to palmitate or GTP indicating that the uncoupled respiration is due to UCP gene function. In addition, disruption of UCP4B/C in *per*^01^ flies reverted other hallmarks of uncoupling: mitochondrial leak respiration (Fig. 3d); mitochondrial membrane potential (Fig. 3e); mitochondrial oxygen consumption rate (Supplementary Fig. 3D); and whole-animal cold shock recovery rates (Fig. 3f). Disruption of the UCP4B/C expression not only reverted mitochondrial uncoupling but also reverted *per*^01^ lifespan to that of controls, suggesting that mitochondrial uncoupling causes lifespan extension (Fig. 3g). To inhibit UCPs by an orthogonal mechanism, we performed *RNAi*-mediated knockdown of UCP4A, B, and C in adulthood throughout the whole body in both *per*^01^ mutants and controls. Consistent with our mutant analysis, knockdown of UCP4B or UCP4C, but not UCP4A, reverted *per*^01^ lifespan to that of control animals (Supplementary Fig. 3E–G). Thus, the Ucp4B/C expression is necessary for the longevity and metabolic phenotypes of *per*^01^ mutants.

Finally, to determine if increased UCP expression is sufficient to extend the lifespan of WT animals, we ubiquitously overexpressed UCP4C at low levels during adulthood using conditional GeneSwitch drivers induced by the drug RU486. Flies overexpressing UCP4C showed mitochondrial uncoupling phenotypes very similar to *per*^01^ mutants; increased mitochondrial leak respiration (Fig. 3h), decreased mitochondrial membrane potential (Fig. 3i), increased CO_2_ production (Supplementary Fig. 4A), and faster cold shock recovery (Supplementary Fig. 4B). Most importantly, the constitutive expression of UCP4C extended the lifespan of otherwise wild-type flies to the same extent as *per*^01^ mutants, with no effect on *per*^01^ mutants (Fig. 3j–k). Feeding these low doses of RU486 alone had no effect on either metabolic or lifespan phenotypes when flies lacked the *UAS* transgene (Supplementary Fig. 3C–G). These results suggest that UCP4C functions in the same pathway as Per to extend *per*^01^ mutant lifespan and that increased expression of the mitochondrial uncoupling protein UCP4C extends lifespan. Taken together, our data point to mitochondrial uncoupling as the major circadian-regulated physiology directly responsible for the extended lifespan of *per*^01^ mutants.

### Lifespan extension is mediated by loss of Per in intestines

**Fig. 4:**
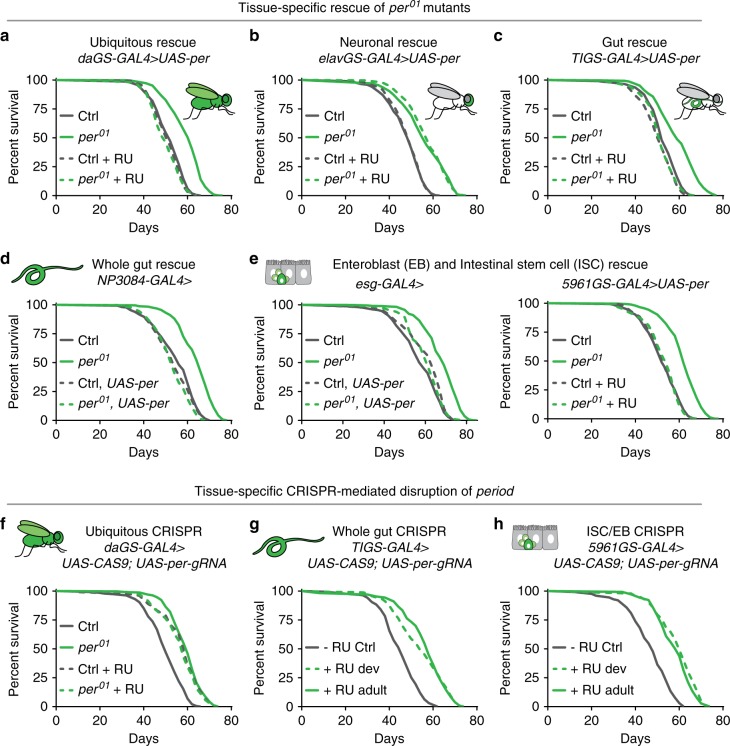
Lifespan extension is mediated by loss of Per specifically in the intestine. Tissue-specific rescue of Per expression (dashed lines) in the *per*^01^ background (green) and controls (gray). **a** Ubiquitous rescue of Per during adulthood was sufficient to revert *per*^01^ lifespan to control levels. While neuronal overexpression of Per (**b**) during adulthood did not revert *per*^01^ lifespan, intestinal overexpression of Per (**c**, **d**) was sufficient to revert *per*^01^ lifespan to control levels. **e** Rescue of Per specifically in intestinal stem cells (ISCs) and enteroblasts (EBs) during development or adulthood reverted *per*^01^ lifespan to control levels. **f** Loss of *period* through ubiquitous CRISPR-mediated deletion during adulthood extended lifespan of otherwise wild-type flies, with no further lifespan extension in *per*^01^ nulls. CRISPR-mediated deletion of *period* in **g** the intestine or **h** IScs and EBs also extended lifespan. See Supplementary Table 1 for *n* and *p* values for lifespan experiments, particularly multicurve comparisons; *p* values were obtained by log-rank analysis.

While circadian sleep/wake cycles are coordinated by a neuronal master clock in the brain, many other circadian functions are regulated by peripheral clocks in specific tissues^30^. To determine whether the master clock or a peripheral circadian clock mediates *per*^01^ longevity, we rescued *period* expression in different organ systems via the *UAS*–*GAL4* system. While ubiquitous expression of Per protein during adulthood reverted the lifespan of *per*^01^ mutants to that of controls (Fig. 4a), neuronal expression of Per did not (Fig. 4b). In contrast, intestinal expression of Per in the whole intestine or specifically in intestinal stem cells (ISCs) and enteroblasts (EBs) reverted *per*^01^ lifespan to that of controls (Fig. 4c–e). This result suggests that loss of Per in the intestine is required for lifespan extension of *per*^01^ mutants.

To directly test if loss of *per* in the intestine is sufficient to extend lifespan, we used a *UAS–GAL4*-based CRISPR system to disrupt the *period* gene^31,32^. Ubiquitous deletion of *per* via the *daughterless* GeneSwitch driver (*daGS–Gal4*) was sufficient to extend lifespan of control flies but not *per*^01^ mutants (Fig. 4f). Similar to *per*^01^ mutants, flies with ubiquitous disruption of *period* were completely arrhythmic in constant darkness (Supplementary Fig. 5A). We then performed tissue-specific CRISPR-mediated disruption of *period* in either the whole intestine or specifically in ISCs/EBs during either development or adulthood and found that any of these manipulations was sufficient to extend lifespan (Fig. 4g, h). Disruption of *period* in the whole intestine or ISCs and EBs had no influence on circadian locomotor activity (Supplementary Fig. 5B, C). To control for CRISPR-mediated DNA damage, we disrupted an unrelated gene involved in sperm storage in females (*acp98AB*)^33^ using the same inducible GAL4 drivers. *acp98AB* disruption did not extend lifespan using any of these tissue-specific drivers (Supplementary Fig. 5D–F). Similarly, RU486 feeding had no influence on control and *per*^01^ mutants lacking *UAS* transgenes (Supplementary Fig. 5G–I). CRISPR-targeted disruption of *period* in ISCs/EBs resulted in >90% reduction in *per* transcript in the intestine after 30 days of induction (Supplementary Fig. 5J). Taken together, these data show that the intestinal circadian clock plays a role in limiting lifespan in *Drosophila*.

### UCP4C in the intestine is necessary for *per* mutant longevity

**Fig. 5:**
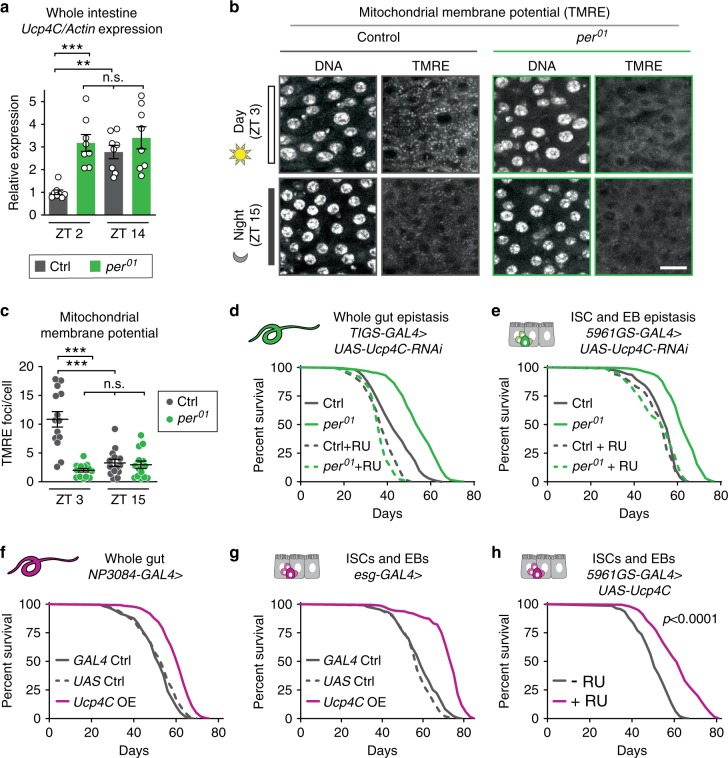
UCP4C in the intestine is necessary for loss of Per-mediated lifespan extension and sufficient to extend wild-type lifespan. **a** Whole intestine expression levels of *Ucp4C* during day and night show oscillations of *Ucp4C* expression in controls with constitutively high expression in *per*^01^ mutants. **b** Representative images of the posterior midgut of control (left) and *per*^01^ mutants (right) stained with Hoechst (DNA) and TMRE during the day and night (scale bar = 30 μm). **c** While wild-type intestines exhibited high membrane potential during the day and low membrane potential at night, *per*^01^ flies exhibited low membrane potential at both times of day. Knockdown of *Ucp4C* in the whole intestine (**d**) or ISCs/EBs (**e**) reverted *per*^01^ lifespan to control levels. Relative to controls (gray), flies overexpressing *Ucp4C* (magenta) in the whole intestine (**f**) or ISCs and EBs (**g**, **h**) had extended lifespan (*p* < 0.0001 for each). See Supplementary Table 1 for *n* and statistical analysis of lifespans; ^n.s.^*p* > 0.05, *p < 0.05, **p < 0.01, ***p < 0.001; *p* values were obtained by ANOVA followed by Tukey’s post-hoc test (**a**, **c**) and log-rank analysis (**d**–**h**); error bars represent SEM.

To determine if mitochondrial uncoupling is circadian-regulated in the intestine, we first measured mRNA levels of *Ucp4C* in dissected whole intestines during the day and night (Fig. 5a). In control intestines, *Ucp4C* was low during the day and elevated at night, while *per*^01^ mutants exhibited constitutively high *Ucp4C* expression during both day and night. To further evaluate circadian regulation of uncoupling activity in the gut, we assessed mitochondrial membrane potential at different times of day in dissected intestines from wild-type flies and *per*^01^ mutants, stained with the dye TMRE, which accumulates in mitochondria with higher membrane potential. Loss of membrane potential indicates increased mitochondrial uncoupling. In controls, intestinal mitochondrial membrane potential was indeed circadian-regulated, with higher membrane potential (lower uncoupling) during the day and lower membrane potential (higher uncoupling) at night (Fig. 5b, c). Consistent with loss of circadian regulation, *per*^01^ mutants exhibited low mitochondrial membrane potential at both timepoints, suggesting high levels of mitochondrial uncoupling during both day and night. This phenotype is not due to changes in mitochondrial abundance, as both controls and *per*^01^ mutants have similar intestinal mitochondrial populations, as measured by mitoGFP fluorescence (Supplementary Fig. 6A, B). Thus, mitochondrial uncoupling in the *Drosophila* intestine is circadian-regulated.

Because we found that ubiquitous UCP4C expression was necessary and sufficient for lifespan extension (Fig. 3), we next tested whether intestine-specific UCP4C expression is necessary and sufficient for lifespan extension. Similar to *per* rescue, *RNAi*-mediated knockdown of UCP4C in the whole intestine or in ISC/EB populations reverted *per*^01^ lifespan to that of controls (Fig. 5d, e). Moreover, while the overexpression of UCP4C in the nervous system did not alter lifespan (Supplementary Fig. 6C), constitutive expression of UCP4C in the whole intestine (Fig. 5f, Supplementary Fig. 6D) or specifically in ISCs and EBs (Fig. 5g, h) resulted in extended lifespan and lowered mitochondrial membrane potential in otherwise wild-type animals (Supplementary Fig. 6E–H). Thus, intestinal UCP4C expression is not only necessary for the lifespan extension of *per*^01^ mutants but also sufficient to extend lifespan in otherwise wild-type *Drosophila*. That is, intestine-specific upregulation of a normally circadian-regulated, oscillating physiological function, mitochondrial uncoupling, extends lifespan.

### Per and UCP4C control intestinal homeostasis via ROS levels

**Fig. 6:**
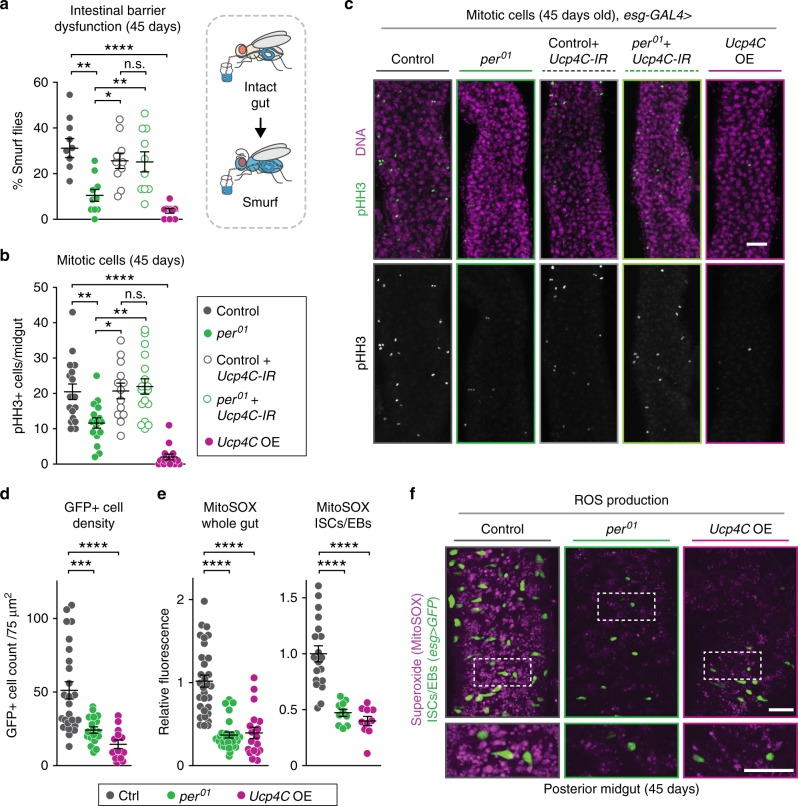
Loss of *period* preserves intestinal homeostasis via increased mitochondrial uncoupling and decreased ROS levels. **a** Smurf assays of 45-day-old flies showed *per*^*01*^ mutants had reduced populations with intestinal barrier dysfunction, a phenotype dependent upon *Ucp4C* expression, and that overexpression of *Ucp4C* also reduced intestinal barrier dysfunction. **b** Quantification of intestinal pHH3+ staining showed that *per*^*01*^ mutants had lower levels of age-related hyperproliferation dependent on UCP4C expression and that overexpression of UCP4C caused a large reduction in mitotic cells (*n* = 13–17 intestines). **c** Representative images of phospho-histone H3 at residue S10 (pHH3) staining of midguts from 45-day flies (scale bar = 40 μm). (**d**, **e**) *per*^01^ mutants and *Ucp4C*-overexpressing flies exhibited delayed *esg* + cell overproliferation (**e**) and lower ROS output of all posterior midgut cells, including ISC/EB populations. **f** Representative images for MitoSOX staining of aged posterior midguts in control *esg-GAL4* > *GFP* flies, *per*^01^*; esg-GAL4* > *GFP*, and *esg-GAL4* > *GFP;UAS-Ucp4C flies* (scale bars = 35 μm (top) and 15 μm (bottom inset location indicated by dashed lines). See Supplementary Table 1 for *n* and statistical analysis of lifespans; ^n.s.^*p* > 0.05, *p < 0.05, **p < 0.01, ***p < 0.001, ****p < 0.0001; *p* values were obtained by ANOVA followed by Tukey’s post-hoc test (**b**, **c**, **e**, **f**); error bars represent SEM.

To understand the underlying mechanism by which mitochondrial uncoupling in the intestine extends lifespan, we tested if loss of *per* or intestinal UCP4C overexpression delayed aging-related defects in the intestine. When flies age, they typically lose intestinal barrier function, which is also a major predictor of mortality^34–37^, suggesting that lifespan can be extended by preventing aging-related intestinal barrier dysfunction. We tested for aging-related intestinal barrier dysfunction using the “smurf assay,” named after a children’s cartoon, which measures leakage of an ingested blue dye^36,37^. Consistent with their extended lifespan, old *per*^01^ mutants showed a lower percentage of “smurfs” relative to controls, indicating a delay in aging-related intestinal barrier dysfunction (Fig. 6a). Moreover, this maintenance of intestinal integrity observed in *per*^01^ mutants depended on intestinal expression of UCP4C and intestinal overexpression of UCP4C alone in otherwise wild-type flies was sufficient to maintain intestinal integrity. Thus, loss of Per and increased mitochondrial uncoupling in the intestine protect against aging-related intestinal dysfunction.

As aging-related intestinal barrier dysfunction is linked to overproliferation and tissue dysplasia in the gut^38^, we next tested if loss of *per* or intestinal UCP4C overexpression delayed aging-related cellular proliferation in the gut. Per protein has previously been shown to regulate ISC regeneration during acute intestinal stress^39^. To measure cellular proliferation in the intestines, we stained aged *per*^01^ and control flies for phospho-histone H3 at serine 10 (pHH3), a standard proliferative marker that labels mitotic cells (Fig. 6b, c). Further consistent with delayed aging, *per*^01^ mutants showed fewer pHH3 positive cells in the gut relative to control flies. To determine if this phenotype depends on mitochondrial uncoupling, we knocked down UCP4C in *per*^01^ flies and controls. UCP4C knockdown reverted the number of proliferative cells in *per*^01^ flies to the higher levels seen in controls. Moreover, the overexpression of UCP4C in otherwise wild-type controls was sufficient to decrease age-related overproliferation of ISCs/EBs. Thus, similar to the smurf assay and consistent with lifespan results, intestinal UCP4C expression is both necessary in *per*^01^ mutants and sufficient in otherwise wild-type controls to suppress aging-related intestinal cellular proliferation.

Because the microbiome and age-related intestinal dysbiosis can contribute to intestinal barrier dysfunction, intestinal dysplasia, and proliferative state^40–42^, we sought to determine if gut bacteria play a role in *period* null longevity.  First, we measured overall microbial load in aging flies using universal 16S bacterial qPCR. Higher bacterial load is associated with aging and mortality. We found that long-lived *per*^01^ flies did not have decreased bacterial load but instead exhibited an increased bacterial load during aging relative to controls (Supplementary Fig. 7A). To determine if this increased microbial load influenced lifespan, we fed control and *per*^01^ flies throughout adulthood an antibiotic cocktail that reduced intestinal bacterial levels to nearly undetectable levels (Supplementary Fig. 7B). Feeding antibiotics for the entire lifespan had no impact on the extended longevity of *per* nulls relative to controls (Supplementary Fig. 7C).  Together, these data show that the microbiome present in our animals did not cause *per*^01^ longevity.

Intestinal dysfunction and increased aging-related cellular proliferation are closely associated with elevated Reactive Oxygen Species (ROS) production. ROS can serve as important mitogenic signals in stem cells and their differentiated progeny cells in the fly^43^; elevated ROS production in the *Drosophila* intestine is thought to lead to increased misdifferentiation of proliferative cells, ISCs, and EBs^44^. Because mitochondrial uncoupling can restrict ROS output^45–47^, we hypothesized that uncoupled *per*^01^ mutants have extended longevity due to decreased mitochondrial ROS production in the intestine, leading to a decrease in age-related overproliferation of *esg*-positive (*esg*+) ISC/EB cells. To test this, we stained the intestines of aged *per*^01^ mutants, flies expressing UCP4C constitutively in ISCs and EBs, and controls with MitoSOX Red, a fluorescent mitochondrial ROS (superoxide) indicator (Fig. 6f). Both *per*^01^ mutants and intestinal UCP4C-expressing flies exhibited reduced *esg*+ populations and decreased mitochondrial superoxide production relative to controls in the whole posterior midgut, as well as in ISC/EB populations (Fig. 6d, e). Together, these results suggest that loss of Per function and increased mitochondrial uncoupling via upregulated UCP4C decrease ROS levels in the intestine.

### Uncoupling drugs extend lifespan via gut homeostasis

**Fig. 7:**
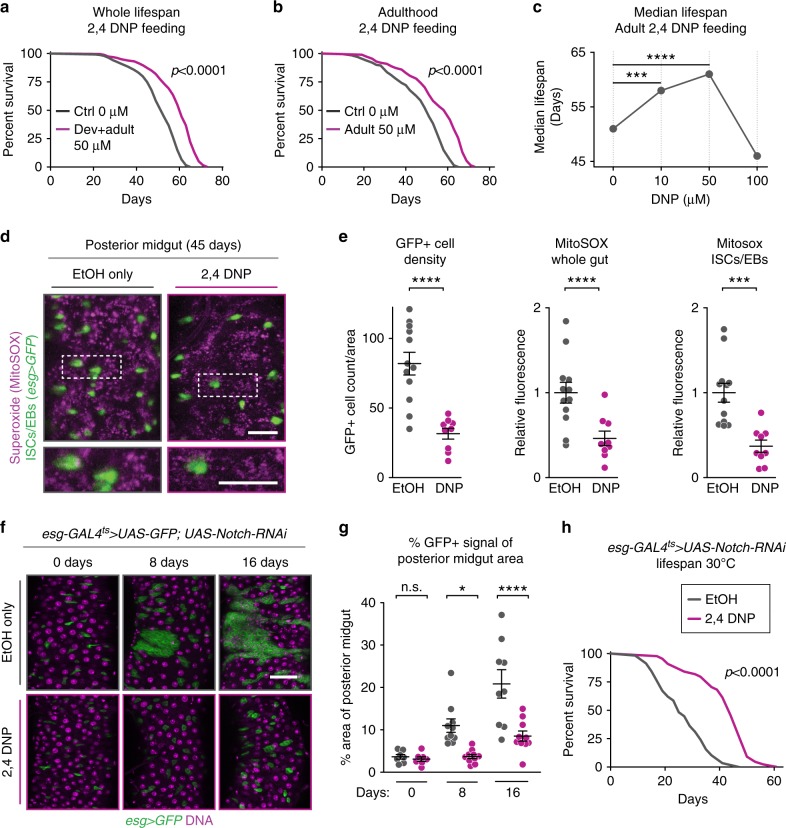
Pharmacological reduction of ROS via uncoupling preserves intestinal homeostasis and extends lifespan. Flies fed the mitochondrial uncoupler 2,4-DNP (magenta) either throughout their lifespan (**a**) or only during adulthood (**b**) showed extended lifespan relative to vehicle controls (gray, *p* < 0.0001 for each). **c** 2,4-DNP feeding increased median lifespan in a dose-dependent manner. **d** Representative images of MitoSOX staining of posterior midguts in *esg-GAL4* > *GFP* flies fed vehicle or DNP (scale bars = 25 μm, top; 10 μm, bottom, inset location indicated by dashed lines). **e** Flies fed 2,4-DNP exhibited: fewer *esg* + cells and thus lower ISC/EB overproliferation with age (left); decreased Mitosox staining of the entire posterior midgut and thus decreased mitochondrial superoxide output (middle); and decreased MitoSOX staining specifically in ISCs/EBs marked by GFP (right). **f** Representative images of midguts from flies exhibiting loss of Notch-mediated tumor formation (scale bar = 35 μm). Relative to vehicle**-**fed flies, flies with Notch-induced tumor formation that were fed 2,4-DNP exhibited delayed tumor formation as measured by GFP + area in the midgut (**g**) and extended lifespans (**h**, *p* < 0.0001 for each). See Supplementary Table 1 for *n* and statistical analysis of lifespans; ^n.s.^*p* > 0.05, *p < 0.05, **p < 0.01, ***p < 0.001, ****p < 0.0001; *p* values were obtained by log-rank analysis (**a**, **b**, **c**, **h**), unpaired two-tailed *t*-test (**e**), and ANOVA followed by Tukey’s post-hoc test (**g**); error bars represent SEM.

To extend our genetic experiments, we tested if pharmacological induction of mitochondrial uncoupling recapitulates the intestinal phenotypes of *per*^01^ mutants and extends lifespan. We tested low dietary concentrations of two compounds, the mitochondrial uncoupling compound 2,4-DNP and uncoupling-agent/antioxidant beta-hydroxytoluene (BHT). To identify the effective dose of these compounds, we first showed that both compounds extended the lifespan of wild-type male flies when used within a range of physiological concentrations (Fig. 7a–c, Supplementary Fig. 6A)^48,49^. To ensure that these drugs did not cause diet restriction, we confirmed that DNP or BHT did not decrease feeding rate (Supplementary Fig. 6B). We found that these drugs did not extend the lifespan of *per*^01^ mutants, suggesting that these mutants are already receiving the maximal benefit of mitochondrial uncoupling (Supplementary Fig. 6C, D). Consistent with the hypothesis that uncoupling-induced lifespan extension is mediated by reduced cellular proliferation and decreased ROS levels, dietary DNP at concentrations that extend lifespan also reduce age-related *esg+* cell overproliferation and mitochondrial ROS output (Fig. 7d, e). Thus, pharmacological induction of mitochondrial uncoupling extends lifespan and recapitulates many of the metabolic phenotypes of *per*^01^ mutants and UCP4C-expressing flies.

To test whether the mitochondrial uncoupling-induced suppression of cellular overproliferation is specific to aging-related cellular overproliferation and misdifferentiation or may act as a general mechanism to suppress cellular overproliferation, we tested the effects of the uncoupling drug DNP on an induced intestinal tumor model. *Notch*-Delta signaling normally keeps proliferative intestinal cells (ISCs/EBs) at a homeostatic level of differentiation and division^50^; loss of Notch in ISCs and EBs causes cellular overproliferation and the formation of ISC-derived intestinal tumors^50,51^. Changes to ISCs that alter differentiation and result in overproliferation are a common step in cancer development^52^. To test if mitochondrial uncoupling could slow tumor progression in the gut, we induced ISC tumors by RNAi suppression of *Notch* signaling, followed by feeding of 2,4-DNP (to induce mitochondrial uncoupling) or vehicle. Dietary 2,4-DNP slowed the progression of ISC tumors (Fig. 7f, g) and extended the short lifespan of these animals (Fig. 7h). These results suggest that pharmacological induction of mitochondrial uncoupling can metabolically suppress stem cell proliferation not only during normal aging but also during tumorigenesis.

## Discussion

Circadian regulators are known to control the organism’s metabolism, including mitochondrial uncoupling;^2,53^ the relationship between circadian regulation, metabolism, and lifespan is highly complex. Here we showed that the male circadian mutant *per*^01^ is long-lived due to a mechanism independent of, and additive with, canonical methods of life extension. Loss of *period* increased mitochondrial uncoupling, resulting in hyperphagic yet lean animals that maintain intestinal health with age. Ablating circadian regulation via loss of Per or increasing circadian-regulated mitochondrial uncoupling specifically in the intestine was sufficient to lower intestinal ROS levels, preserve proliferative homeostasis and gut barrier function, and extend lifespan. These beneficial intestinal phenotypes were recapitulated in wild-type flies pharmacologically by feeding low doses of two different mitochondrial uncoupling drugs. Finally, 2,4-DNP feeding was also capable of delaying tumor formation in a model of genetically induced ISC overproliferation. Thus, while mitochondrial uncoupling has previously been proposed to extend lifespan and healthspan, we demonstrate here an underlying mechanism related to intestinal tissue homeostasis^45,49,54,55^. Our overarching hypothesis posits that loss of Per and circadian rhythms leads to a state of increased mitochondrial uncoupling, which decreases ROS production in the intestine (Supplementary Fig. 9). This prevents aging-related stem cell overproliferation and misdifferentiation, resulting in lifespan extension and, in the case of genetically induced tumors in *Drosophila*, inhibition of tumor growth.

Our initial finding that loss of Per extends the lifespan of *Drosophila* is controversial, as other groups reported that *per* null mutants have shorter lifespan than controls^5,8,9^. We observed this short lifespan phenotype only with *per* males fed very high protein content diets. Environmental factors such as diet composition were strictly controlled in our experiments. Furthermore, we confirmed our findings using novel CRISPR techniques to delete *period* from the whole organism and specifically in the intestine (Fig. 4), resulting in robust lifespan extension. Contradictions in the field have been attributed in part to differences in pathogen status or the microbiome^10,12^. Upon microbiome removal via antibiotic feeding, we still observed significant lifespan extension in *period* nulls, indicating that the microbiome found in our flies does not contribute to this phenotype (Supplementary Fig 7). Regional and diet-based differences in the microbiome can significantly alter lifespan of the organisms^56^. Our experiments do not rule out a more pathogenic microbiome in some environmental conditions that could preferentially shorten *period* mutant lifespan. Consistent with this, *per* mutants display varying susceptibility and resistance to pathogenic infection^16,19,57,58^. In *Drosophila*, the composition of the microbiome can greatly influence the inflammation level (including ROS production) and the resulting proliferative status of the intestine^59,60^. Further investigation into the intestinal microbiome composition of circadian mutants and resulting inflammation and immune response or other environmental factors could shed light onto these findings.

The molecular mechanism underlying circadian regulation of mitochondrial UCP expression remains unknown. Consistent with previous findings that circadian regulators such as PER2 and REVERBα can regulate the expression of uncoupling protein UCP1 in mice^61,62^, we showed that the expression of mitochondrial uncoupling proteins UCP4C/B is circadian-regulated in wild-type *Drosophila* and constitutively high in *period* nulls (Fig. 3). However, there are no canonical Clock-binding E-box sequences present in the promoter regions of these genes, suggesting that Clock and Cycle do not directly regulate UCP4C/B expression. Thus, we predict that Per disruption causes the accumulation of an unidentified transcription factor that directly regulates UCP expression; this will be an area of active future investigation. Moreover, UCPs are activated posttranslationally by specific fatty acid components and lipid metabolism is altered in *per* mutants^8^. Additional understanding of the circadian regulation of UCP expression and activity could support the benefits of metabolic treatments such as intermittent fasting or different treatment schedules for antidiabetic drugs such as metformin, as well as other therapeutics that regulate metabolism and lifespan.

Our main hypothesis posits that mild mitochondrial uncoupling mitigates ROS production in aged mitochondria, which is consistent with previous reports^45,46^. This mild reduction in proton gradient may trade energy inefficiency with the benefit of reduced ROS output. While the precise mechanism by which a mildly reduced proton gradient mitigates ROS production remains unclear, it is known that high ROS production is correlated with increased membrane potential^46,47^. UCPs could serve to mitigate this potentially damaging state throughout the circadian cycle as energy demands change with time of day.

This UCP-lowered ROS production in the intestine is sufficient to maintain proliferative homeostasis in the gut (Fig. 6). Our data also support the hypothesis that ROS or ROS-induced signals sensed by ISCs/EBs cause their proliferation and contribute to dysregulated tissue homeostasis in the gut. We found that UCP4C overexpression in ISCs/EBs alone is sufficient to lower ROS output of the whole posterior midgut, including fully differentiated enterocyte cells (ECs), not only the ISC/EB population from which ECs are derived. Future work will investigate how this UCP-mediated lowered ROS production is passed on from ISCs/EBs to differentiated daughter ECs, the major cell type of the intestine. While it is possible that ECs inherit this low ROS metabolic state via UCPs in EB mitochondria, this phenotype could also be due to non-cell-autonomous signaling from ISCs/EBs to surrounding ECs. The mechanism(s) by which ISC/EBs pass on this metabolic state via uncoupling may be a diffusible signal, such as mitochondrially generated ROS itself, metabolic rewiring due to altered metabolite utilization, or a new type of “mitokine” that is currently undiscovered. Further investigation into how mitogenic signals are coordinated between cell-types of the intestine may reveal the mechanism by which this metabolic state is maintained. In addition, tight regulation of mitochondrial metabolism and oxidative state is conserved in the process of mammalian ISC differentiation^63,64^. Thus, our work supports a role for oxidative stress in the maintenance of ISC identity with age, a role likely to be evolutionarily conserved.

Our use of mitochondrial uncoupling compounds such as 2,4-DNP showed that they can recapitulate lifespan extension in *Drosophila*. 2,4-DNP was originally used in high doses in the 1930s as weight loss supplements or diet pills^65^, but dangerous side-effects such as hyperthermia, cataracts, and cardiac dysfunction led to its discontinuation. More recent findings in mice^49,66^ suggest that very low doses can reduce body fat and potentially increase lifespan, but the tissue-specific mechanism by which this compound acts to increase longevity remained elusive. Here we provide evidence that 2,4-DNP limits ROS output of the intestine, thus preserving intestinal homeostasis with advanced age and even delaying tumor growth (Fig. 7). Together, these data suggest that mitochondrial uncoupling induces a metabolic state that inhibits cellular proliferation. In yeast, mitochondrial uncoupling is known to signal to the nucleus and cause profound changes in gene expression^67^; future investigation will determine if this is true for multicellular organisms and if these changes in gene expression cell-autonomously inhibit proliferation of stem cells. Our data provide insight into circadian-regulated mitochondrial function to improve intestinal healthspan and organismal longevity and implicate mitochondrial uncoupling as a potential therapeutic to control stem cell overproliferation/misdifferentiation and delay aging.

## Methods

### Fly strains

*cycle* (*cyc*^01^) *period* (*per*^01^) and *timeless* (*tim*^01^) mutants outcrossed to the *Canton-S* (CS) background as controls were obtained from Jaga Giebultowicz. *pBAC-Ucp4C*^e03988^*, UAS-DN-S6K* (6911) were obtained from the Bloomington Stock Center. *UAS-Ucp4A-RNAi, UAS-Ucp4B-RNAi,* and *UAS-Ucp4C-RNAi* were obtained from the Vienna Drosophila Resource Center. *UAS-Ucp4C* was received from flyORF. *UAS-GFP-Atg8* from Eric Baehrecke, *ubi-GAL4* from Marc Dionne, *esg-GAL4, NP3084-GAL4*, *daughterless-Gene-Switch (daGS), elav-Gene-Switch* and *TIGS-2* (*TIGS-GAL4*), *5961GS-GAL4*, *dilp2-GAL4* and *UAS-Reaper* from David W. Walker, *UAS-per10 and UAS-per24*, and *UAS-DN-Clock* from Amita Sehgal, *esg*^*ts*^*-GAL4*, and *UAS-Notch-RNAi* were from Ben Ohlstein. All experiments with multiple transgenes used flies that have undergone 12 generations of out-crossing into a *w*^*1118*^ *CS control and/*or *per*^01^*, w*^*1118*^ *CS* mutant background.

### UAS-CRISPR line construction

Multiple gRNAs targeting *per*, or *acp98AB* were constructed according to (Port and Bullock, *Nature Methods*, 2016). In short, *pCFD6* (Addgene #73915) was digested with BbsI-HF (NEB #R3539S) and the linearized plasmid gel purified. For each construct, inserts were generated in three separate PCR reactions using *pCFD6* as the template and the primers listed below. The resulting three inserts and the pCFD6 backbone were then assembled by NEBuilder HiFi DNA Assembly (NEB #E2621L) for each construct. Each construct was then micro-injected at integration site *Su(Hw)attP5* (Bestgene Inc.) and Sanger sequenced.

UAS-per-gRNA, guide RNA targets:

1. GCTTTTCTACACACACCCGG

2. CACGTGCGATATGATCCCGG

3. GGAGTCCACACACAACACCA

4. TACTCGTCCATAGACCACGC

*per_PCR1fwd*-GCGGCCCGGGTTCGATTCCCGGCCGATGCAGCTTTTCTA

CACACACCCGGGTTTTAGAGCTAGAAATAGCAAG

*per_PCR1rev-* CCGGGATCATATCGCACGTGTGCACCAGCCGGGAATCGAACCC

*per_PCR2fwd-*CACGTGCGATATGATCCCGGGTTTTAGAGCTAGAAATAGCAAG

*per_PCR2rev-*TGGTGTTGTGTGTGGACTCCTGCACCAGCCGGGAATCGAACCC

*per_PCR3fwd-*GGAGTCCACACACAACACCAGTTTTAGAGCTAGAAATAGCAAG

*per_PCR3rev*- ATTTTAACTTGCTATTTCTAGCTCTAAAACGCGTGGTCTATGGACGAGT

ATGCACCAGCCGGGAATCGAACCC

UAS-Ctrl-gRNA, guide RNA targets:

1. GTGTCCCCTTATTCGTGCGG

2. CACACTATCAAAGGATGACG

3. ATAAGGGGACACACTATCAA

4. AGTGTGTCCCCTTATTCGTG

*acp98AB_PCR1fwd*-GCGGCCCGGGTTCGATTCCCGGCCGATGCAGTGTCCCCTTAT

TCGTGCGGGTTTTAGAGCTAGAAATAGCAAG

*acp98AB_PCR1rev*-CGTCATCCTTTGATAGTGTGTGCACCAGCCGGGAATCGAACCC

*acp98AB_PCR2fwd-*CACACTATCAAAGGATGACGGTTTTAGAGCTAGAAATAGCAAG

*acp98AB_PCR2rev-*TTGATAGTGTGTCCCCTTATTGCACCAGCCGGGAATCGAACCC

*acp98AB_PCR3fwd-*ATAAGGGGACACACTATCAAGTTTTAGAGCTAGAAATAGCAAG

*acp98AB_PCR3rev-*ATTTTAACTTGCTATTTCTAGCTCTAAAACCACGAATAAGGGGA

CACACTTGCACCAGCCGGGAATCGAACCC

### Fly media

*Drosophila* were reared from embryos in low-density bottles with standard medium containing 3.8% glucose, 1.9% sucrose, 3% yeast nutritional flake (Lab Scientific), 6.5% cornmeal, 0.8% agar, supplemented with 1.5% methylparaben mix (10% methylparaben in ethanol), and 1% propionic acid. Adult flies that eclosed within a 24 h period were collected and transferred to “adult medium” containing 3.8% glucose, 1.9% sucrose, 8% cornmeal, 1% agar, and either 0.01, 0.5, 3, 5, or 10% YE (Difco) supplemented with 1.5% methylparaben mix and 1% propionic acid for lifespan and biochemical analysis. All percentages given in wt/v except methylparaben mix, propionic acid given in v/v.

### Drug supplementation in media for lifespan

All drugs were supplemented into cooled (65 °C) liquid adult medium (containing 3% YE) following preparation to the following final concentration(s): RU486 (Cayman Chemical) was dissolved in ethanol was supplemented into medium after eclosion at a final concentration of 100 μg/mL (with the exception of *daGS>UAS-Ucp4c* and controls, 5 μg/mL) vehicle controls were supplemented with same volume of ethanol alone. Developmental induction was achieved by adding RU486 at concentration of 5 μg/mL, or ethanol vehicle, followed by adults being aged on standard food containing no RU486. For microbiota clearance medium contained 500 μg/ml ampicillin, 50 μg/ml tetracycline, and 200 μg/ml rifamycin in 50% ethanol. Same volume of 50% ethanol alone was used as the vehicle control. For uncoupler feeding 2,4-DNP or BHT purchased from Sigma was dissolved in ethanol fresh at the time of making food and added into the media, at the indicated concentrations. 2,4-DNP (0.01–0.1 mM) BHT (0.1–1 mM), the same volume of ethanol alone was used as vehicle control.

### Lifespan analysis and starvation

Newly eclosed flies (~24 h) were collected and allowed to mate for 48 h. Female and male flies were separated and maintained at a density of 30–35 flies per vial in a humidified, temperature-controlled (25 °C) incubator with a 12-h light–dark (LD) cycle. For starvation analysis, flies were aged for 7 days in vials containing indicated diets and then transferred into 1% agar in a humidified, temperature-controlled incubator with 12-h LD cycle at 25 °C. Death was scored every 1–2 days, with live flies transferred into fresh vials every 2–3 days. Statistical significance was determined by log-rank analysis.

### Western blot analysis

Whole-body lysates of 10-day-old male flies (30 flies/sample/timepoint) were separated by SDS-PAGE using standard procedures. Membranes were probed with antibodies against AMPK phospho-T184 at 1:1000 (Cell Signaling, 40H9); anti-phospho-S6K T398 (Cell Signaling, 9209), anti-GFP (Cell Signaling, D5.1), antiphospho AKT s505 (Cell Signaling, 4054), and pan AKT at 1:1000 (Cell Signaling, 9272); and horseradish peroxidase (HRP)-conjugated monoclonal mouse antiactin antibody at 1:5000 (Sigma-Aldrich, A3854). Rabbit antibodies were detected using HRP-conjugated anti-rabbit IgG antibodies at 1:2000 (Cell Signaling, 7074). Mouse antibodies were detected using HRP-conjugated anti-mouse IgG antibodies 1:2000 dilution (Cell Signaling, 7076). ECL chemiluminescence reagent (Pierce) was used to visualize horseradish peroxidase activity and detected by CCD camera using a Kodak Image Station. A minimum of four independent samples of each condition were used for statistical analysis and quantification.

### Quantitative real-time PCR

Thirty whole male flies, or ten whole intestines per biological replicate were used for total RNA extraction via TRIzol reagent (Invitrogen) following manufacturer protocols. Samples were treated with DNase, and then cDNA was synthesized by the Revertaid First Strand cDNA Synthesis Kit (Thermo Scientific). A minimum of four independent samples were used for statistical analysis and quantification. For 16s bacterial rDNA quantification 10 whole flies (washed twice in 70% ethanol, and twice in PBS)  were used for total DNA extraction via the Power Soil DNA isolation kit (Mo Bio). Universal primers for the 16S ribosomal RNA gene were against variable regions 1 (V1F) and 2 (V2R), as previously published^68^.

Equalized amplicons of *Actin5C* were used as a reference gene to normalize expression via the following primer sets:

*Ucp4A-*fwd- TTTGACTACGCGGACTCATTC

*Ucp4A-*rev- CGCGGTATTGCATATTGGACTT

*Ucp4B-*fwd- AACACAGTCTTTAGGCCAGCA

*Ucp4B-*rev- CCGTGAGGTAGAGTTCAACCG

*Ucp4C-*fwd- ACAAACGTCGCTGATCCACTA

*Ucp4C-*rev- GGAAGACACACGACTCGGC

*period*-fwd- GGTTGCTACGTCCTTCTGGA

*period*-rev- TGTGCCTCCTCCGATATCTT

*16Sr*-fwd- AGAGTTTGATCCTGGCTCAG

*16Sr*-rev- CTGCTGCCTYCCGTA

*Actin5C*-fwd- TTGTCTGGGCAAGAGGATCAG

*Actin5C*-rev- ACCACTCGCACTTGCACTTTC

### Cold shock recovery assay

Seven to ten days old males were anesthetized on ice and individually placed in vials and loaded into a DAM5 activity monitor. Flies were maintained at 4 °C for 1 h, and then placed in a 25 °C incubator and allowed to recover for 4 h. Individual flies were scored as recovered at the time three beam breaks were recorded by the activity monitor system. A minimum of 22 flies per condition were used for statistical analysis (log-rank analysis).

### Quantification of triglycerides levels

Lipids were extracted from five whole flies in a chloroform:ethanol solution (2:1 vol/vol), and nonpolar lipids (fatty acid, triacylglycerol) were separated by thin-layer chromatography with an n-hexane/diethylether/glacial acetic acid solution (70:30:1, vol/vol/vol). Plates were air-dried and stained (with 0.2% Amido Black 10B in 1 M NaCl), and lipid bands were quantified by photo densitometry using ImageJ software. Densitometry values were normalized to the mass of the homogenized flies used in the sample following a reference standard of pre-measured coconut oil or butter. A minimum of six independent samples were used for statistical analysis and quantification.

### Intestinal barrier dysfunction assay

The "smurf fly"/intestinal barrier dysfunction assay was performed similarly to ref. ^36^. Flies were aged on regular medium until the day of the smurf assay. Dyed medium was prepared by the addition of FD&C Blue No. 1 at a final concentration of 2.5% wt/vol. A fly was counted as a smurf when dye coloration was observed outside the digestive tract. Comparisons of smurf proportion per timepoint were carried out using binomial tests to calculate the probability of having as many smurfs in population A as in population B, as well as ANOVA for proportions of smurf flies per replicate vial with a minimum of 7 vials of 15–31 flies per replicate.

### Feeding assay

Analysis of capillary feeding (“the CAFE assay”) was performed similarly to ref. ^69^ with minor modifications. Briefly, ten flies were placed in vials with wet tissue paper as a water source and a capillary food source (3.8% glucose, 1.9% sucrose, 3% YE, and 0.2% FD&C Blue No. 1). Feeding was monitored for at least 24 h, replacing depleted capillaries as necessary. A minimum of eight groups of ten flies per condition were used for each experiment.

### CO_2_ respiration assay

Measurement of CO_2_ production was performed similarly to ref. ^70^. Briefly, ten flies were placed in a sealed pipette tip-capillary respirometer (without anesthesia) containing a small amount of soda lime. Respirometers were placed capillary side down in dH_2_0 containing 0.1% blue food dye in a sealed TLC chamber placed in a 25 °C incubator. Empty sealed respirometers (containing no flies) were used to subtract blue dye background migration from experimental (fly-containing) samples. Flies were tested for 2 h between at circadian timepoints, and CO_2_ production rate was indirectly determined by the amount of liquid movement up the capillary per hour after background subtraction. At least six groups of ten flies were used per genotype for quantification for each condition. For mitochondrial drug feeding experiments, flies were placed on food containing the following compounds for 24 h: 500 μM Oligomycin, 1 mM rotenone, 5 mM 2,4-DNP, 0.5% stearate, or ethanol vehicle controls.

### Mitochondrial purification for downstream assays

Fifty whole flies were gently crushed in chilled mitochondrial isolation medium (MIM) (250 mM sucrose, 10 mM Tris-HCl (pH 7.4), 0.15 mM MgCl_2_) passed through a 40 μm filter basket and spun twice at 1000 × *g* for 5 min at 4 °C to remove nuclei and debris. The supernatant was then spun at 4000× g, for 10 min at 4 °C. The pellet, containing the mitochondria, was washed once in fresh MIM buffer and then total protein content of the resulting pellet was determined by the Bradford Assay.

### Mitochondrial oxygen consumption measurements

Protein content of purified mitochondria was normalized, and samples were resuspended in modified MiR05 buffer (0.5 mM EGTA, 3 mM MgCl_2_, 60 mM K-lactobionate, 20 mM taurine, 10 mM KH_2_PO_4_, 20 mM HEPES, 110 mM sucrose, and 0.25 g/L BSA, pH 7.2). Mitochondrial respiration was measured at 25 °C using high-resolution respirometry (Oxygraph-2k, Oroboros). To determine max ADP stimulated respiration initiated through complexes I and II, the following sequential injections were made (final concentration in chamber): 5 mM glutamate plus 5 mM malate, 5 mM succinate, 1 mM ADP. To determine nonphosphorylating leak respiration, 3 µM Oligomycin was then added. To measure reserve electron transport capacity of the mitochondria, carbonyl cyanide m-chlorophenyl hydrazine (CCCP) 0.5 mM stock was titrated into the chamber at 1 µL increments. Residual nonmitochondrial oxygen consumption was determined by the addition of 1 µM rotenone, and 2.5 µM antimycin A. Respiration rates of mitochondria initiated through complexes I, II, and IV were determined by the following injection/reaction schema (final concentrations in chamber): 5 mM glutamate plus 5 mM malate, 1 mM ADP, 0.5 µM rotenone, 5 mM succinate, 2.5 µM antimycin A, and 1 mM ascorbate plus 0.25 mM N′-tetramethyl-1,4-phenylenediamine, 0.5 mM KCN. A minimum of four independent mitochondrial preps were run for each condition. For determination of palmitate stimulated respiration and inhibition by GTP mitochondria were washed three times in modified MiR05 containing 1% fatty acid free BSA to remove any free fatty acids present. Respiration was stimulated through complex II by the addition of 5 mM succinate in the presence of 0.5 µM rotenone. BSA-conjugated to palmitate (Agilent) was added to a final Palmitate concentration of 10 nM, followed by 2 mM GTP (Thermo), and finally 2.5 µM antimycin A. A minimum of three independent mitochondrial preps were run for each condition.

### Membrane potential JC-1 assay

Protein content of purified mitochondria was normalized, and samples were resuspended at a concentration of 200 µg/mL in modified MiR05 buffer (0.5 mM EGTA, 3 mM MgCl_2_, 60 mM K-lactobionate, 20 mM taurine, 10 mM KH_2_PO_4_, 20 mM HEPES, 110 mM sucrose, and 0.25 g/L BSA, pH 7.2) containing 20 µg/mL JC-1 dye. (488ex/530em) and (533ex/590em) spectra were monitored via plate reader at 25 °C. To measure membrane potential at steady-state ATP production stimulated through complexes I and II: 5 mM glutamate plus 5 mM malate, 5 mM succinate, 1 mM ADP were added to the preparation. To determine membrane potential upon inhibited ATP synthesis, 3 µM oligomycin was then added. To further control for the function of the dye, 25 µM of CCCP was then added to dissipate the proton gradient. A minimum of four independent mitochondrial preps were run for statistical analysis.

### Blue NativePAGE and in-gel activity assay

Assays were performed as in ref. ^71^. Mitochondria were purified from ten thoraces of 7–10-day-old male flies and BN-PAGE was performed using NativePAGE gels from Life Technologies, following the manufacturer’s instructions. Mitochondria were suspended in NativePAGE sample buffer (Life Technologies) supplemented with 1% digitonin and protease inhibitors, incubated on ice for 20 min and centrifuged at 20,000 × *g* for 30 min at 4 °C, the supernatant was recovered, G-250 (Life Technologies) sample additive and NativePAGE Sample Buffer was added before loading onto 3–12% precast Bis–Tris NativePAGE gels (Life Technologies). Electrophoreses was performed using the NativePAGE Running buffer (as anode buffer, from Life technologies) and the NativePAGE Running buffer containing 0.4% Coomassie G-250 (cathode buffer). Protein complexes were revealed by staining with Novex Colloidal Blue staining kit (Life Technologies). Complex I activity in native gels was performed by incubating gels in 0.1 mg/mL NADH, 2.5 mg/mL nitrotetrazolium blue chloride, 5 mM Tris-HCl (pH 7.4) overnight at room temperature. Gels were imaged using a BioRad station and densitometry was performed in ImageJ.

### Circadian locomotor activity

Post eclosion, flies were reared in a humidified, temperature-controlled incubator with 12-h LD cycle at 25 °C. Seven-day-old adults were anesthetized under light CO_2_ and placed individually into Drosophila Activity Monitor (DAM) tubes, containing standard food. Flies were monitored using a DAM2 system (Trikinetics) for 3 days in 12-h LD cycle for circadian entrainment, followed by 6 days of constant darkness (DD) to assess free running locomotor activity. ClockLab (Coulbourn Industries) was used to determine the rhythmicity and period of the fly populations in constant darkness. A minimum of 16 flies per genotype were used for each experiment.

### TMRE/MitoSOX intestinal staining

Flies were anesthetized on ice and intestines were dissected in cold Schneider’s Medium (Thermo Fisher Scientific). Intestines were then incubated in 75 nM of TMRE (Thermo Fisher Scientific) in Schneider’s Medium, for 10 min at room temperature. Samples were rinsed three times for 30 s with a solution consisting of 20 nM of TMRE Schneider’s Medium. For MitoSOX staining dissected intestines were immersed in 50 µM MitoSOX Red (Invitrogen) in Schneider’s Medium for 5 min at room temperature, and then washed three times for 30 s in Schneider’s Medium. MitoSOX or TMRE samples were quickly mounted in Schneider’s Medium containing 1.5 µg/mL Hoechst stain. Intestines were imaged within 20 min on a Ziess LSM800 confocal microscope. *Z*-stacks spanning the entire posterior midgut were taken. The quantification of TMRE or MitoSOX was performed using ImageJ in which mean fluorescent intensity values were quantified. TMRE foci were quantified using the ImageJ^72^ local maxima tool, using identical thresholding for all images. Foci number were normalized to the number of nuclei in the measured field of view. The quantification of MitoSOX output of ISCs/EBs was determined by mean intensity from individual *Z*-planes with regions of interest defined by esgGFP expression, cell size, and basal location within the intestinal epithelium. Mean intensity of MitoSOX fluorescence levels in all ISCs/EBs were averaged for each individual intestine. A minimum of nine midguts were used for each genotype or drug treatment.

### Phospho-histone H3 immunostaining

Briefly, flies were anesthetized on ice and intestines were dissected in cold PBS. Samples were then fixed in PBS + 0.1% Triton X-100 containing 4% paraformaldehyde at room temperature for 30 min and rinsed three times in PBS + 0.1% Triton X-100 for 10 min at room temperature. Blocking was performed in 5% BSA in PBS + 0.1% triton X-100 for 1 h at room temperature. Primary antibody, anti-phospho-histone H3 (S10) (Cell Signaling, 9701), was added 1:250 in 5% BSA in PBS + 0.1% triton X-100 and incubated overnight at 4 °C rotating. After washing three times in PBS + 0.1% Triton X-100 secondary antibody, anti-rabbit AlexaFluor-488 (Invitrogen) was added 1:250, and 1.5 µg/mL Hoechst stain (Thermo) in 5% BSA in PBS + 0.2% triton X-100 and incubated overnight at 4 °C rotating. After washing, intestines were then mounted in Vectashield mounting medium (Vector Labs) and Imaged using Zeiss Ziess LSM800. pHH3 positive cells were quantified using the ImageJ^72^ local maxima tool, with identical thresholding for all images. pHH3 numbers were normalized to the area of the posterior midgut imaged. A minimum of ten intestines were used for each quantification.

### esg^ts^>UAS-Notch-RNAi intestine staining

*esg*^*ts*^*>UAS-Notch-RNAi* flies were reared at 18 °C. Upon eclosion flies were collected, sexed, and placed on media containing 0.05 mM 2,4-DNP, or ethanol vehicle food at 30 °C. Timepoints of 0, 8, and 16 days were taken. Intestines were dissected, fixed, and stained as above pHH3 protocol, with anti-GFP (Cell Signaling, D5.1) and 1.5 µg/mL Hoechst stain. The quantification of GFP area was performed by using the ImageJ count particle tool in the GFP channel normalized to total area of the posterior midgut that was imaged. Seven to thirteen guts were used for each condition and timepoint.

### Statistical analysis

Prism7 (GraphPad) was used to perform the statistical analysis significance is expressed as *p* values (^ns^*p* > 0.05, *p < 0.05, **p < 0.01, ***p < 0.001). For two group comparisons, unpaired, two-tailed *t*-test was used, when data met criteria for parametric analysis (normal distribution and similar variance). Mann–Whitney *U*-test was used in case of nonparametric analysis. For more than two group’s comparison, ANOVA with Bonferroni or Tukey’s post-hoc test was performed. Kruskal–Wallis with dunn’s post-hoc for data that was not distributed appropriately for the parametric comparisons. For comparison of survival curves, Log-rank (Mantel–Cox) test was used.

### Reporting summary

Further information on research design is available in the Nature Research Reporting Summary linked to this article.

## Supplementary information

Supplementary Information

Reporting Summary

## Data Availability

The authors declare that all data supporting the findings of this study are available within the paper and its Supplementary Information files. The data for all graphs shown in this paper are available in source data file. Additional raw data, and replicate experiments will be made available upon reasonable request.
