# Supplementary material for: Circadian regulation of mitochondrial uncoupling and lifespan
Source: Nat Commun. 2020 Apr 21;11:1927. doi: 10.1038/s41467-020-15617-x (PMC7174288; doi:10.1038/s41467-020-15617-x)
Supplement: Supplementary file 1 — Supplementary Information [file 41467_2020_15617_MOESM1_ESM.pdf]

Description of Supplementary files

File Name: Source Data
Description: Source data for all graphs presented in this paper.

File Name: Supplementary information
Description: Supplementary Figures, legends, and Supplementary Table

Supplementary Figure 1, related to Figure 1

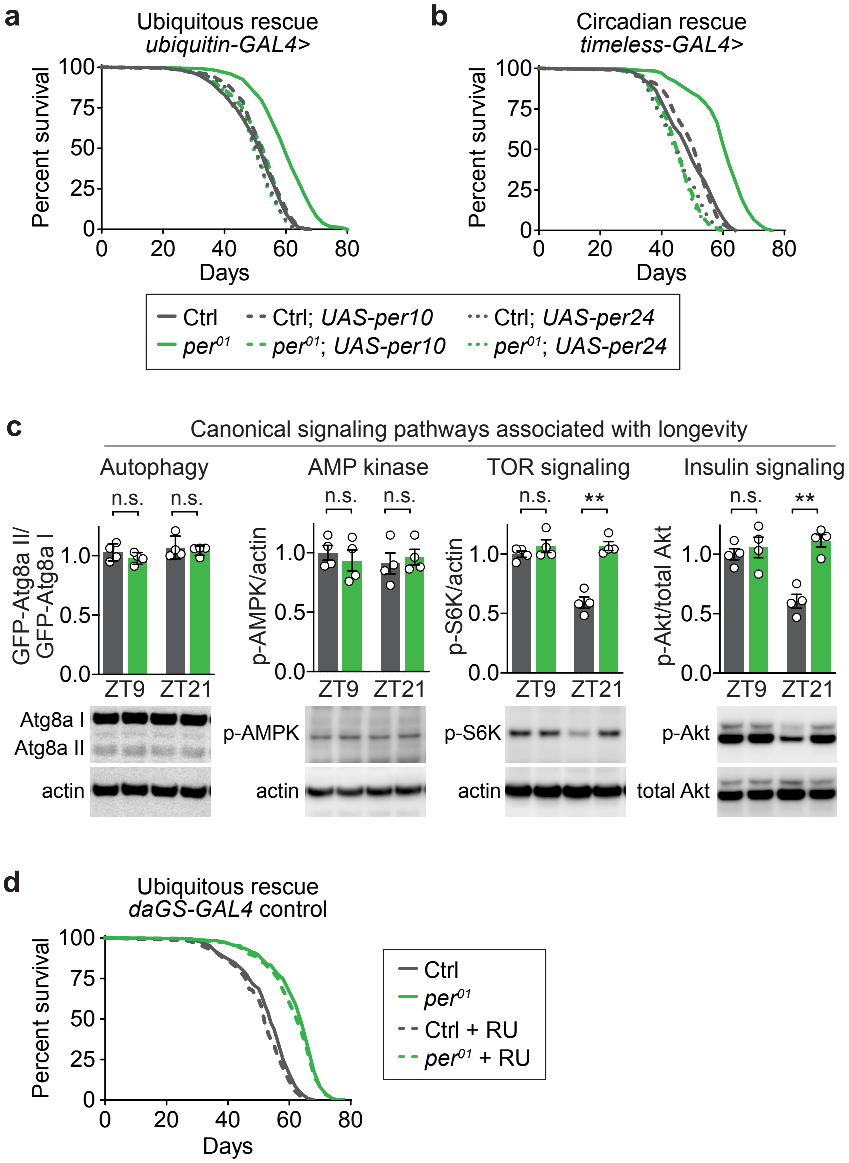

Supplementary Figure 1. Exogenous rescue of Period in *per*^{01} null mutants, Western blot analysis of canonical longevity pathways, and RU486 lifespan controls. (a, b) Rescue of Per expression in *per*^{01} null mutants using either *UAS-per24* or *UAS-per10* transgenes via *ubiquitin-GAL4* **(a)** or *timeless-GAL4* **(b)** reverted *per*^{01} lifespan back to that of wild-type flies. **(c)** Western blot analyses of longevity-associated pathways performed at different circadian time points (n=4 samples per condition): phosphorylation of Akt at serine 505, of S6K at threonine 398, of AMPK at threonine 184, and lipidation of GFP-tagged Atg8a. **(d)** Feeding of RU486 (dashed lines) or vehicle (solid lines) in *per*^{01} mutants and controls containing *daGS-GAL4* drivers lacking UAS-transgenes did not influence lifespan. See Supplementary Table 1 for n and statistical analysis of lifespans; n.s. = $p>0.05$, * = $p<0.05$, **= $p<0.01$, *** = $p<0.001$, **** = $p<0.0001$; p-values were obtained by log-rank analysis (a, b, d) and ANOVA followed by Tukey's post-hoc test (c); error bars represent SEM.

Supplementary Figure 2, related to Figure 2

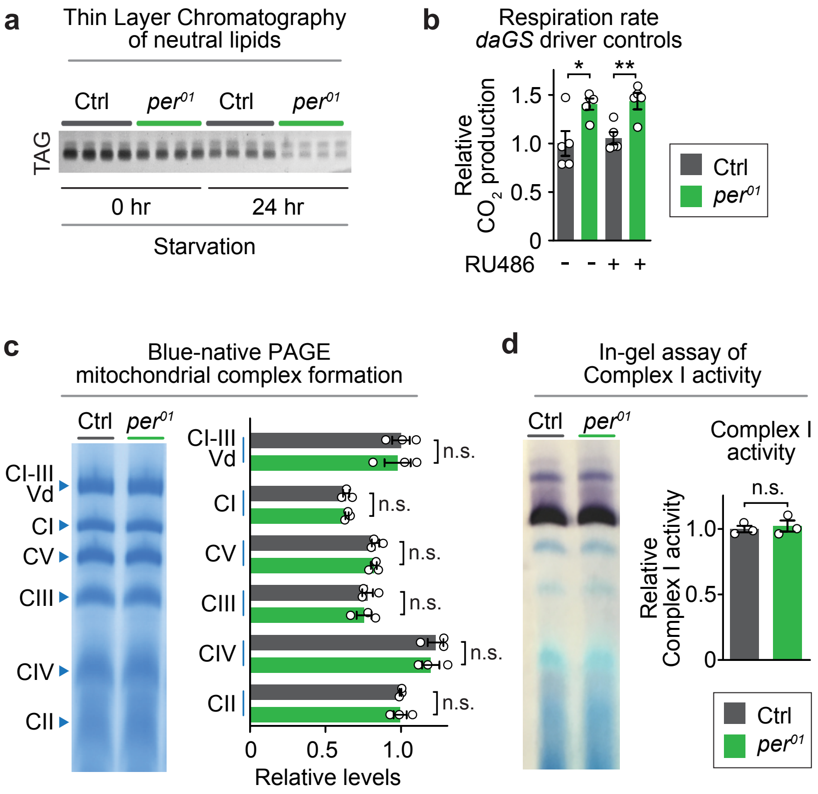

Supplementary Figure 2. ***per*** **mutants exhibit decreased lipid stores, rapid lipid depletion during starvation, no change in respiration with RU486 treatment, and normal levels of mitochondrial proteins and activity. (a)** Representative images of thin-layer chromatography of TAG for quantification of triacylglyceride (TAG) levels in per^{01} mutants and controls (n≥4 samples/condition, 5 flies/sample) showed that per^{01} flies exhibited lower levels of lipids compared to control flies at baseline (0 hr) and increased rate of lipid utilization after 24 hours of starvation. **(b)** Feeding RU486 in flies lacking UAS-transgenes did not alter respiration rates in either mutants or controls (n=6 groups of 10 flies per condition, ANOVA followed by Tukey's post-hoc test). **(c)** Blue-native PAGE of purified mitochondria from mutants and controls with quantification of complex formation (right) showed that per^{01} and control lines had no difference in specific electron transport chain complex abundance (n=3 mitochondrial preps, p>0.05 for all comparisons). **(d)** In-gel activity assay for complex I from mutants and controls with quantification (right) showed that per^{01} and control lines had no difference in complex I activity (n=3 mitochondrial preps, p>0.05 for all comparisons). Statistical significance: n.s. = p>0.05, * = p<0.05, **= p<0.01, *** = p<0.001, **** = p<0.0001; p-values were obtained by ANOVA followed by Tukey's post-hoc test (a) and two-tailed, unpaired t-test (c, d); error bars represent SEM.

Supplementary Figure 3, related to Figure 3

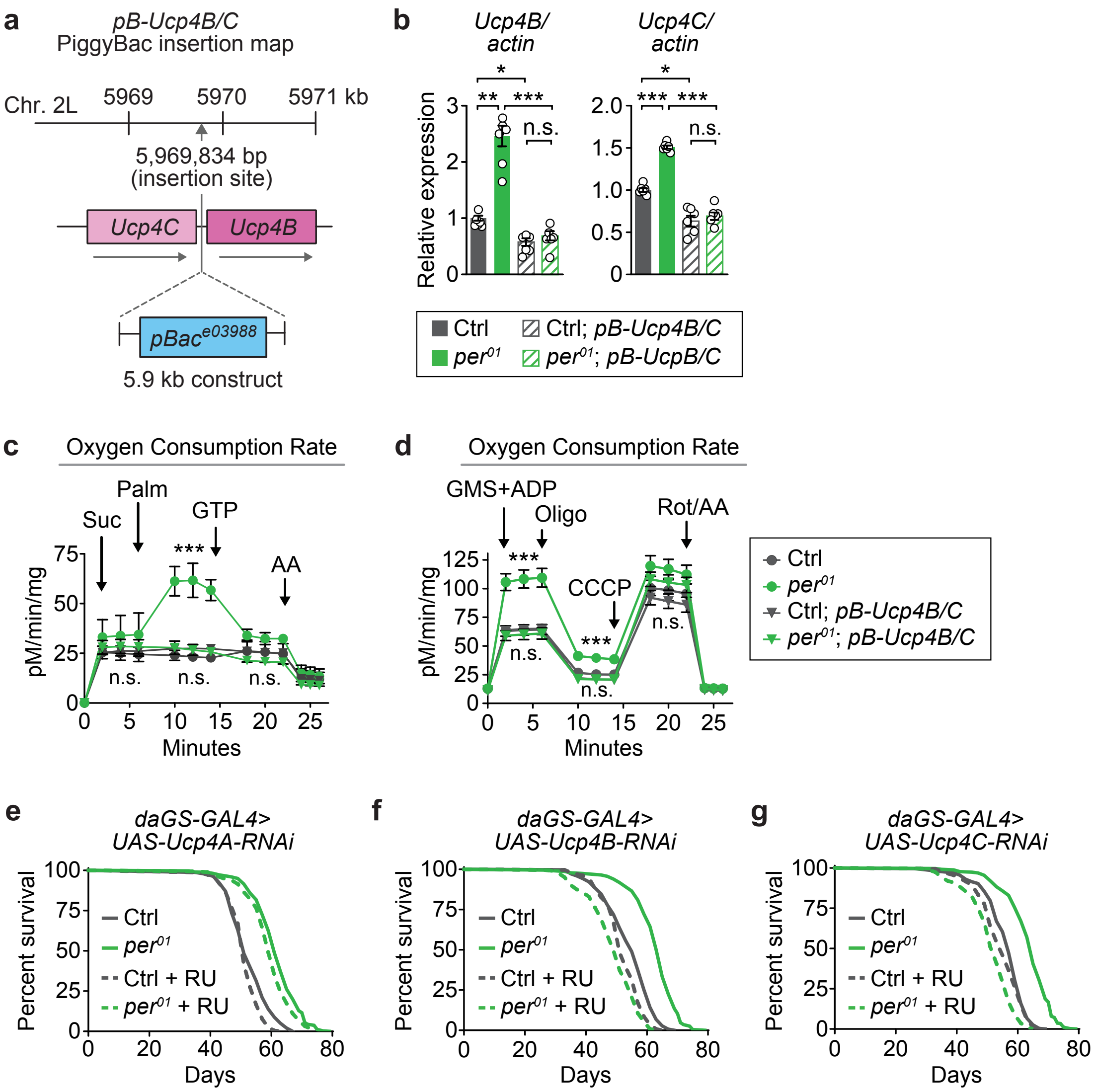

Supplementary Figure 3. Mitochondrial uncoupling of *per*^{01} mutants are dependent on *Ucp4B* or *Ucp4C* expression and UCP4C overexpression causes mitochondrial uncoupling phenotypes (a) Schematic representation of piggyBAC insertion disrupting intergenic region between *Ucp4B* and *Ucp4C*. **(b)** Expression levels of *Ucp4B/C* were reduced to near wild-type levels in *per*^{01} nulls harboring pBAC mutation (n=6 biological samples pooled from 6 individual circadian timepoints per condition). **(c)** High-resolution respirometry of mitochondria purified from *per*^{01} and control lines with or without piggyBac mutation of *Ucp4B/C* showed that *per*^{01} mutant mitochondria exhibited palmitate dependent stimulation of respiration which is reversed by the addition of GTP indicating high uncoupling protein activity. This activity was abolished by disruption of *Ucp4B/C* expression (n=3 mitochondrial preps per condition). (**d**) Respirometry on mitochondria purified from *per*^{01} and control lines with or without piggyBac mutation of *Ucp4B/C* showed that *per*^{01} mutant mitochondria exhibited higher respiration rates under steady-state ATP production and oligomycin inhibited leak state respiration, phenotypes abolished by suppression of *Ucp4B/C* activity (n=4-6 mitochondrial preps per condition). **(e-g)** While induced ubiquitous knockdown of *Ucp4A* **(e)** did not alter the difference between control and *per*^{01} lifespans, induced knockdown of *Ucp4B* **(f)** and *Ucp4C* **(g)** significantly reduced *per*^{01} lifespan similar to that of controls. See Supplementary Table 1 for lifespan statistical analysis, n.s. = p>0.05, * = p<0.05, **= p<0.01, *** = p<0.001, **** = p<0.0001; p-values were obtained by ANOVA followed by Tukey's post-hoc test (b-d), log-rank analysis (e-g); error bars represent SEM.

Supplementary Figure 4, related to Figure 3

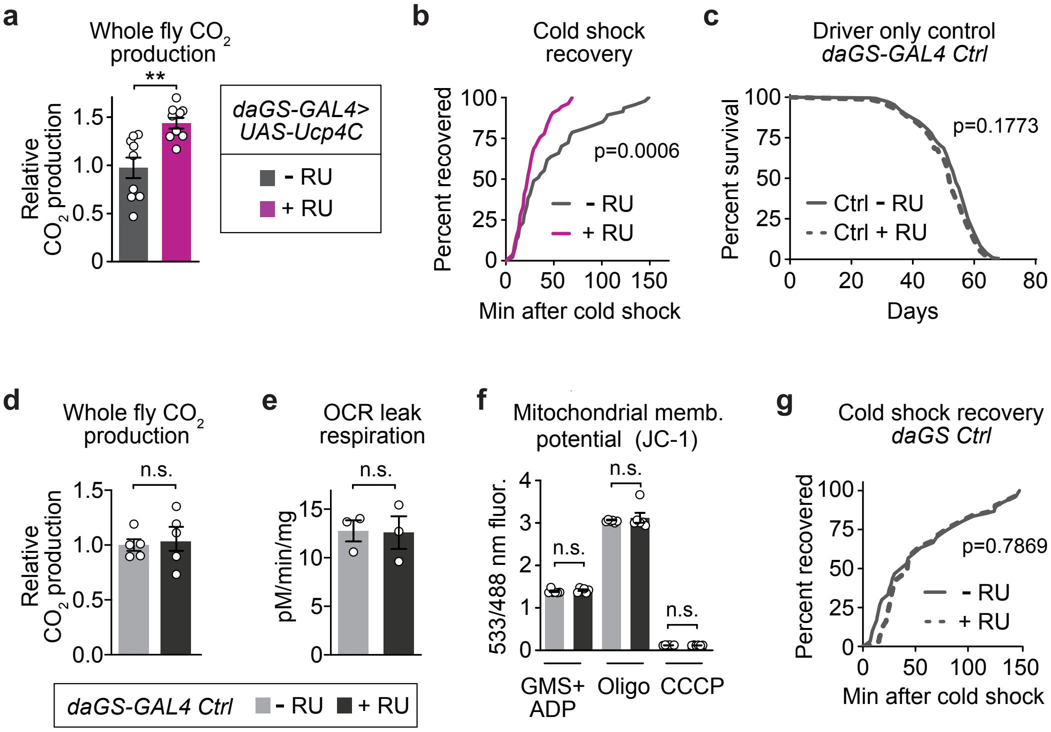

Supplementary Figure 4. RU486 feeding alone has no influence on lifespan and mitochondrial uncoupling phenotypes. (a-b) Ubiquitous overexpression of UCP4C resulted in increased respiration (**a**, n≥8 groups of 10 flies per condition, p<0.01) and faster cold shock recovery (**b** n=47-54 flies per condition, p<0.001). **(c-e)** Feeding of RU486 during adulthood to flies containing *daGSS-GAL4* drivers but lacking UAS transgenes did not extend lifespan **(c)** or alter: respiration rates (**d,** n=5 groups of 10 flies per condition); leak state oxygen consumption (**e,** n=3 oxygraph runs per condition); mitochondrial membrane potential (**f**, n=6 mitochondrial preps per condition); or recovery from cold shock in control lines (**g**, n=24-25 flies per condition, p=0.723). See Supplementary Table 1 for lifespan statistical analysis, n.s. = p>0.05, * = p<0.05, **= p<0.01, *** = p<0.001, **** = p<0.0001; p-values were obtained by unpaired two-tailed t-test (a, d-f), and log-rank analysis (b, g); error bars represent SEM.

Supplementary Figure 5, related to Figure 4

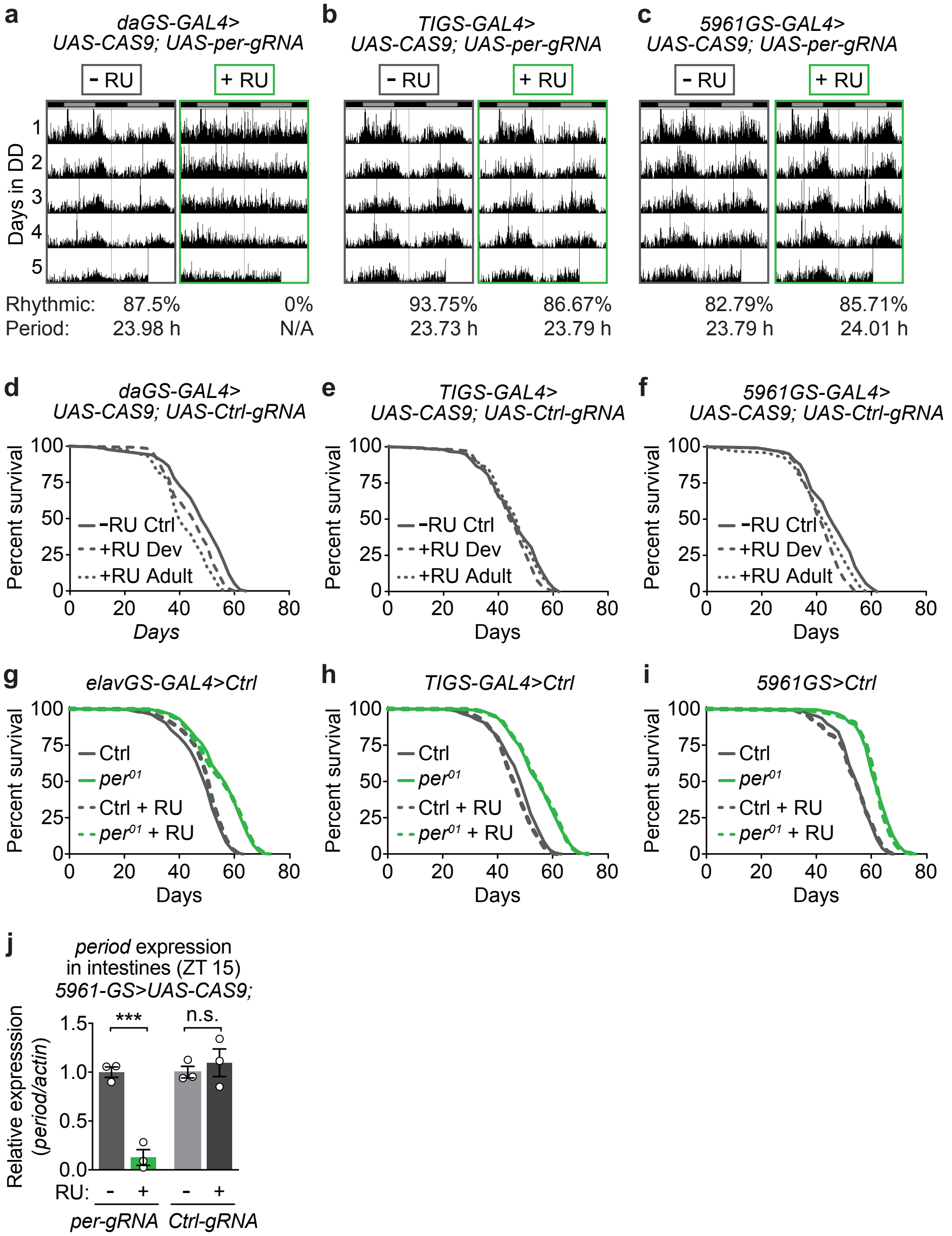

Supplementary Fig. 5. Lifespan controls for CRISPR-mediated DNA damage; RU486 feeding does not influence lifespan of control or *per*^{01} mutants with tissue-specific drivers lacking UAS-transgenes. (a) Actograms of flies with developmentally induced ubiquitous deletion of *period* show arrhythmicity in constant darkness. **(b, c)** Flies with intestinal deletion of period show no locomotor rhythmicity defects in constant darkness. **(d-f)** Adulthood-induced CRISPR deletion of a control gene (*acp98AB)* ubiquitously **(d)**, in intestines **(e)**, and in ISC/EB populations **(f)** did not extend the lifespan of male flies. **(g-i)** Feeding of RU486 during adulthood to flies lacking UAS-transgenes but containing neuronal *elavGS-GAL4* **(g)**, intestinal *TIGS-GAL4* **(h)**, or ISC/EB-specific *5961GS-GAL4* **(i)** drivers did not revert *per*^{01} lifespans to control levels nor extend control lifespans. **(j)** Adulthood-induced CRISPR deletion of *period* in ISC/EB populations by feeding of RU486 to flies containing *5961GS-GAL4>UAS-Cas9; per-gRNA* reduced *per* transcript level in the intestine by more than 90% (n=3 samples p<0.001), while adulthood-induced CRISPR deletion of *acp98AB* in ISC/EB populations did not alter *per* transcript levels (n=3 samples 10 intestines/samples, p<0.934). See supplementary Table 1 for lifespan statistical analysis, n.s. = p>0.05, * = p<0.05, **= p<0.01, *** = p<0.001, **** = p<0.0001; p-values were obtained by log-rank analysis (d-i) and unpaired two-tailed t-test (j); error bars represent SEM.

Supplementary Figure 6, related to Figure 5

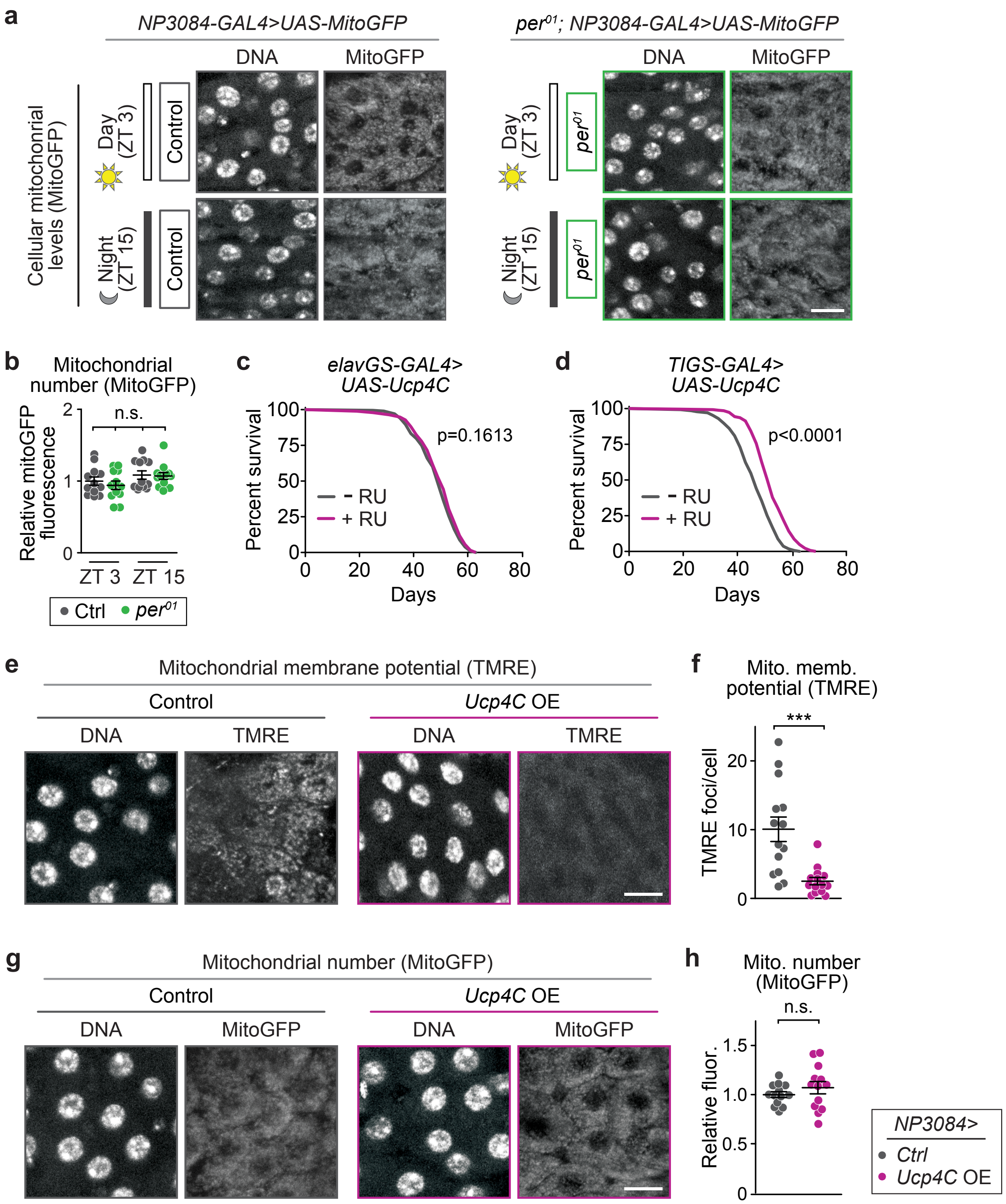

Supplementary Figure 6. Induced mitochondrial uncoupling influences membrane potential and lifespan. (a) Representative images of control and *per*^{01} posterior midguts expressing mito-GFP (scale bar=10μm). **(b)** Quantification of mitochondrial fluorescence from controls and *per*^{01} mutants during day or night showed no significant changes in mitochondrial abundance (n=12 intestines per condition, ANOVA, p=0.2524). **(c, d)** While overexpression of UCP4C in neurons **(c)** caused no lifespan increase (n.s.), overexpression of UCP4C in the intestine **(d)** extended lifespan (p<0.0001). **(e)** Representative images of TMRE staining in posterior midguts from *NP3084-GAL4>+* controls and *NP3084-GAL4>UAS-Ucp4C* flies (scale bar=10μm). **(f)** Quantification of TMRE fluorescence in intestines showed that UCP4C overexpression in the whole intestine (*NP3084-GAL4>UAS-Ucp4C* flies) lowered mitochondrial membrane potential in the posterior midgut relative to controls (*NP3084-GAL4>+,* n=15 intestines per condition, p<0.01). **(g)** Representative images of control and UCP4C-overexpressing posterior midguts labeled by mito-GFP (scale bar=10 μm). **(h)** Quantification of mitochondrial fluorescence (mito-GFP) showed no difference between control and UCP4C overexpressing flies (n=13 intestines per condition, p=0.313). See Supplementary Table 1 for lifespan statistical analysis, n.s. = p>0.05, * = p<0.05, **= p<0.01, *** = p<0.001, **** = p<0.0001; p-values were obtained by ANOVA followed by Tukey's post-hoc test (b), log-rank analysis (c, d), and unpaired two-tailed t-test (f, h); error bars represent SEM.

Supplementary Figure 7, related to Figure 6

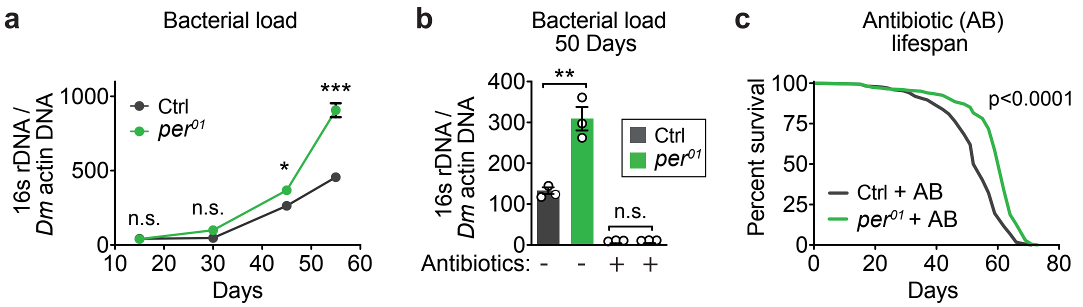

Supplementary Figure 7. *period* mutants exhibit high microbiome load with age and removal of microbiota via antibiotics does not alter *per*^{01} longevity compared to controls. (a) *per*^{01} flies show increased bacterial accumulation with age compared to control animals as marked by universal 16S bacterial PCR (n=4 groups of 20 flies per timepoint). **(b)** Antibiotic treatment effectively renders flies axenic as marked by 16S PCR (n=3 groups of 20 flies per condition)**. (c)** Antibiotic treatment does not alter *per*^{01} lifespan extension (p<0001; log-rank). See Supplementary Table 1 for lifespan statistical analysis, n.s. = p>0.05, * = p<0.05, **= p<0.01, *** = p<0.001, **** = p<0.0001; p-values were obtained by two tailed t-test (a) and ANOVA followed by Tukey's post-hoc test (b); error bars represent SEM.

Supplementary Figure 8, related to Figure 7

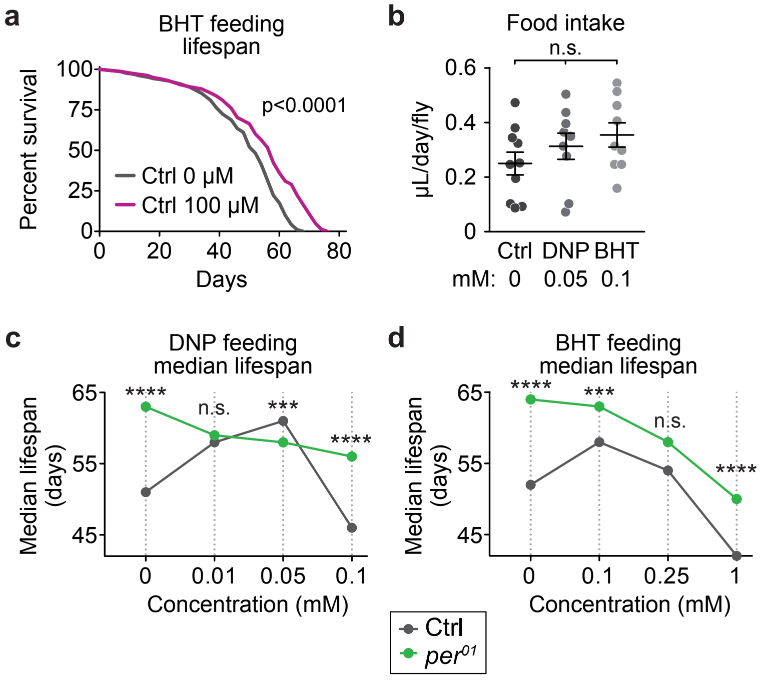

Supplementary Figure 8. Influence of mitochondrial uncoupling on intestinal phenotypes and lifespan. (a) Flies fed the uncoupling compound butylated hydroxyl toluene (BHT) during adulthood had increased lifespan compared to vehicle-fed controls. **(b)** There was no significant difference in feeding rates of flies fed either uncoupling compound 2,4 DNP or BHT relative to ethanol vehicle (n=9-10 groups of 10 flies, p=0.258)**. (c)** While feeding varying concentrations of 2,4 DNP during adulthood to control flies increased their median lifespan in a dose-dependent manner, no median lifespan increase was observed in *per*^{01} null mutants. **(d)** While feeding BHT to control flies increased their lifespan in a dose-dependent manner, no lifespan increase was observed in *per*^{01} nulls. See Supplementary Table 1 for lifespan statistical analysis, n.s. = p>0.05, * = p<0.05, **= p<0.01, *** = p<0.001, **** = p<0.0001; p-values were obtained by log-rank analysis (a, c, d) and ANOVA followed by Tukey's post-hoc test (b); error bars represent SEM.

Supplementary Figure 9

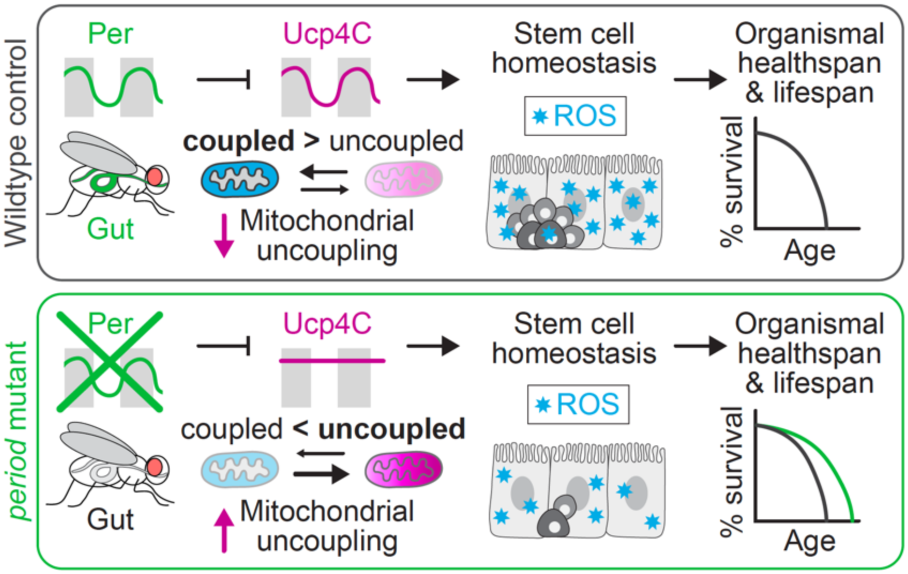

Supplementary Figure 9. Schematic representation of Per and UCP4C influence on longevity and tissue homeostasis. In wild-type animals (upper panel), Per controls the oscillatory expression of UCP4C, leading to oscillation between states of coupled mitochondrial respiration and uncoupled mitochondrial respiration in the intestine over the circadian day. As a result, wild-type animals produce levels of ROS in the gut that promote ISC overproliferation and tissue dysplasia with age and limit lifespan. In contrast, in *per* null mutants (lower panel), loss of Per leads to constitutive upregulation of UCP4C expression and constitutively increased levels of uncoupled mitochondrial respiration. ROS production is limited in the intestine of these animals, resulting in better tissue homeostasis and increased longevity.

Supplementary Table 1. Summary of Lifespan experiments presented in figures

Figure 1 Genotype	Figure	rep	Drug/Diet	n	Median Lifespan	comparison	% change	log-rank p-value
CS control	1B	1	-	98	56			
CS; CS; cyc01	1B	1	-	107	40	ctrl vs. cyc01	-28.57	<0.0001
CS control		2	-	205	60			
CS; CS; cyc01		2	-	111	42	ctrl vs. cyc01	-30.00	<0.0001
daGS>UAS-DN-clk	1C	1	etOH	264	53			
daGS>UAS-DN-clk	1C	1	100μg/ml RU486	454	8	no RU / +RU	-84.91	<0.0001
daGS>UAS-DN-clk		2	etOH	299	50			
daGS>UAS-DN-clk		2	100μg/ml RU486	296	13	no RU / +RU	-74.00	<0.0001
CS control	1F	1	0.01% YE	162	42			
CS; tim01; CS	1F	1	0.01% YE	219	49	ctrl / tim01	16.67	<0.0001
CS control	1F	2	-	348	41			
CS; tim01; CS	1F	2	-	263	53	ctrl / tim01	29.27	<0.0001
CS control	1F	3	-	367	42			
CS; tim01; CS	1F	3	-	308	52	ctrl / tim01	23.81	<0.0001
CS control	1F	1	0.5% YE	378	49			
CS; tim01; CS	1F	1	0.5% YE	346	55	ctrl / tim01	12.24	<0.0001
CS control	1F	2	-	278	53			
CS; tim01; CS	1F	2	-	223	62	ctrl / tim01	16.98	<0.0001
CS control	1F	3	-	404	52			
CS; tim01; CS	1F	3	-	427	60	ctrl / tim01	15.38	<0.0001
CS control	1F	1	3% YE	235	44			
CS; tim01; CS	1F	1	3% YE	358	55	ctrl / tim01	25.00	<0.0001
CS control	1F	2	-	264	48			
CS; tim01; CS	1F	2	-	222	55	ctrl / tim01	14.58	<0.0001
CS control	1F	3	-	334	47			
CS; tim01; CS	1F	3	-	297	56	ctrl / tim01	19.15	<0.0001
CS control	1D/F	4	-	365	46			
CS; tim01; CS	1D/F	4	-	491	58	ctrl / tim01	26.09	<0.0001
CS control	1F	1	10% YE	185	42			
CS; tim01; CS	1F	1	10% YE	254	39	ctrl / tim01	-7.14	<0.001
CS control	1F	2	-	208	45			
CS; tim01; CS	1F	2	-	306	38	ctrl / tim01	-15.56	<0.0001
CS control	1F	3	-	247	42			
CS; tim01; CS	1F	3	-	328	37	ctrl / tim01	-11.90	<0.0001
w1118,CS control	1G	1	0.01% YE	295	43			
per01,w1118;CS;CS	1G	1	0.01% YE	221	50	ctrl / per01	16.28	<0.0001
w1118,CS control	1G	2	-	157	43			
per01,w1118;CS;CS	1G	2	-	70	48	ctrl / per01	11.63	<0.0001
w1118,CS control	1G	3	-	190	43			
per01,w1118;CS;CS	1G	3	-	201	48	ctrl / per01	11.63	<0.0001
w1118,CS control	1G	1	0.5% YE	302	57			
per01,w1118;CS;CS	1G	1	0.5% YE	245	64	ctrl / per01	12.28	<0.0001
w1118,CS control	1G	2	-	154	55			
per01,w1118;CS;CS	1G	2	-	124	68	ctrl / per01	23.64	<0.0001
w1118,CS control	1G	3	-	234	57			
per01,w1118;CS;CS	1G	3	-	267	68	ctrl / per01	19.30	<0.0001
w1118,CS control	1G	1	3% YE	202	48			
per01,w1118;CS;CS	1G	1	3% YE	239	61	ctrl / per01	27.08	<0.0001
w1118,CS control	1G	2	-	137	55			
per01,w1118;CS;CS	1G	2	-	94	62	ctrl / per01	12.73	<0.0001
w1118,CS control	1G	3	-	204	47			
per01,w1118;CS;CS	1G	3	-	218	59	ctrl / per01	25.53	<0.0001
w1118,CS control	1E/G	4	-	543	50			
per01,w1118;CS;CS	1E/G	4	-	551	62	ctrl / per01	24.00	<0.0001
w1118,CS control	1G	1	10% YE	244	43			
per01,w1118;CS;CS	1G	1	10% YE	225	38	ctrl / per01	-11.63	<0.0001
w1118,CS control	1G	2	-	132	43			
per01,w1118;CS;CS	1G	2	-	86	41	ctrl / per01	-4.65	<0.001
w1118,CS control	1G	3	-	170	43			
per01,w1118;CS;CS	1G	3	-	227	38	ctrl / per01	-11.63	<0.0001
Control; daGS>UAS-DN-S6K		1	etOH	176	42	Ctrl no RU / per01 no RU	33.33	<0.0001
Control; daGS>UAS-DN-S6K		1	100μg/ml RU486	187	52	no RU / +RU	23.81	<0.0001
per01; daGS>UAS-DN-S6K		1	etOH	178	56	Ctrl + RU/ per01 + RU	26.92	<0.0001
per01; daGS>UAS-DN-S6K		1	100μg/ml RU486	138	66	per01 no RU / per01 +RU	17.86	<0.0001
Control; daGS>UAS-DN-S6K	1H	2	etOH	199	43	Ctrl no RU / per01 no RU	27.91	<0.0001
Control; daGS>UAS-DN-S6K	1H	2	100μg/ml RU486	253	51	no RU / +RU	18.60	<0.0001
per01; daGS>UAS-DN-S6K	1H	2	etOH	170	55	Ctrl + RU/ per01 + RU	23.53	<0.0001
per01; daGS>UAS-DN-S6K	1H	2	100μg/ml RU486	338	63	per01 no RU / per01 +RU	14.55	<0.0001
Control; dilp2-GAL4>		1	-	149	52	Ctrl / per01	19.23	<0.0001
Control; dilp2-GAL4>UAS-reaper		1	-	145	73	Ctrl / dilp>rpr	40.38	<0.0001
per01; dilp2-GAL4>		1	-	224	62	Ctrl>rpr / per01 ;>rpr	13.70	<0.0001
per01; dilp2-GAL4>UAS-reaper		1	-	160	83	per01 / per01; dilp>rpr	33.87	<0.0001
Control; dilp2-GAL4>	1I	2	-	192	50	Ctrl / per01	24.00	<0.0001
Control; dilp2-GAL4>UAS-reaper	1I	2	-	214	72	Ctrl / Ctrl-dilp>rpr	44.00	<0.0001
per01; dilp2-GAL4>	1I	2	-	219	62	Ctrl>rpr / per01 >rpr	16.67	<0.0001
per01; dilp2-GAL4>UAS-reaper	1I	2	-	281	84	per01 / per01; dilp>rpr	35.48	<0.0001

Figure 3<br>Genotype	Figure	rep	Drug/Diet	n	Median Lifespan	comparison	% change	log-rank p-value
Control	3G	1	-	233	55	Ctrl / per01	18.18	<0.0001
Control; pBAC-Ucp4B/C	3G	1	-	432	52	Ctrl / Ctrl-pBAC	-5.45	<0.001
per01	3G	1	-	270	65	per01-pBAC / Ctrl-pBAC	0.00	ns
per01; pBAC-Ucp4B/C	3G	1	-	457	52	per01 / per01-pBAC	-20.00	<0.0001
Control		2	-	312	53	Ctrl / per01	20.75	<0.0001
Control; pBAC-Ucp4B/C		2	-	126	53	Ctrl / Ctrl-pBAC	0.00	ns
per01		2	-	250	64	per01-pBAC / Ctrl-pBAC	1.89	ns
per01; pBAC-Ucp4B/C		2	-	112	54	per01 / per01-pBAC	-15.63	<0.0001
daGS>UAS-Ucp4C	3J	1	etOH	512	49			
daGS>UAS-Ucp4C	3J	1	5µg/ml RU486	298	57	no RU / +RU	16.33	<0.0001
daGS>UAS-Ucp4C		2	etOH	281	51			
daGS>UAS-Ucp4C		2	5µg/ml RU486	298	60	no RU / +RU	17.65	<0.0001
Control; ubi-GAL4>	3K	1	-	184	50	Ctrl, ubi / per01, ubi	24.00	<0.0001
Control; ubi-GAL4>UAS-Ucp4C	3K	1	-	220	60	Ctrl, ubi/ Ctrl, ubi>UCP4c	20.00	<0.0001
per01; ubi-GAL4>	3K	1	-	221	62	per01, ubi>UCP4c/ Ctrl,ubi>UCP4c	0.00	ns
per01; ubi-GAL4>UAS-Ucp4C	3K	1	-	157	60	per01, ubi / per01, ubi>UCP	-3.23	ns
Control; ubi-GAL4>		2	-	338	51	Ctrl, ubi / per01, ubi	15.69	<0.0001
Control; ubi-GAL4>UAS-Ucp4C		2	-	547	60	Ctrl, ubi/ Ctrl, ubi>UCP4c	17.65	<0.0001
per01; ubi-GAL4>		2	-	315	59	per01, ubi>UCP4c/ Ctrl,ubi>UCP4c	0.00	ns
per01; ubi-GAL4>UAS-Ucp4C		2	-	564	60	per01, ubi / per01, ubi>UCP4C	1.69	ns

Figure 4<br>Genotype	Figure	rep	Drug/Diet	n	Median Lifespan	comparison	% change	log-rank p-value
Control; daGS>UAS-per24	4A	1	etOH	314	52	Ctrl no RU / per01 no RU	19.23	<0.0001
Control; daGS>UAS-per24	4A	1	100µg/ml RU486	323	52	Ctrl no RU / Ctrl +RU	0.00	ns
per01; daGS>UAS-per24	4A	1	etOH	439	62	Ctrl + RU/ per01 + RU	-3.85	<0.05
per01; daGS>UAS-per24	4A	1	100µg/ml RU487	318	50	per01 no RU / per01 +RU	-19.35	<0.0001
Control; daGS>UAS-per24		2	etOH	173	50	Ctrl no RU / per01 no RU	16.00	<0.0001
Control; daGS>UAS-per24		2	100µg/ml RU488	192	50	Ctrl no RU / Ctrl +RU	0.00	ns
per01; daGS>UAS-per24		2	etOH	221	58	Ctrl + RU/ per01 + RU	0.00	ns
per01; daGS>UAS-per24		2	100µg/ml RU489	149	50	per01 no RU / per01 +RU	-13.79	<0.0001
Control; daGS>UAS-per10		1	etOH	141	53	Ctrl no RU / per01 no RU	16.98	<0.0001
Control; daGS>UAS-per10		1	100µg/ml RU490	151	54	Ctrl no RU / Ctrl +RU	1.89	<0.05
per01; daGS>UAS-per10		1	etOH	218	62	Ctrl + RU/ per01 + RU	-3.70	<0.01
per01; daGS>UAS-per10		1	100µg/ml RU491	179	52	per01 no RU / per01 +RU	-16.13	<0.0001
Control; elav>UAS-per24	4B	1	etOH	294	50	Ctrl no RU / per01 no RU	14.00	<0.0001
Control; elav>UAS-per24	4B	1	100µg/ml RU486	272	50	Ctrl no RU / Ctrl +RU	0.00	ns
per01; elav>UAS-per24	4B	1	etOH	268	57	Ctrl + RU/ per01 + RU	14.00	<0.0001
per01; elav>UAS-per24	4B	1	100µg/ml RU487	299	57	per01 no RU / per01 +RU	0.00	ns
Control; elav>UAS-per24		2	etOH	287	50	Ctrl no RU / per01 no RU	16.00	<0.0001
Control; elav>UAS-per24		2	100µg/ml RU488	190	50	Ctrl no RU / Ctrl +RU	0.00	ns
per01; elav>UAS-per24		2	etOH	406	58	Ctrl + RU/ per01 + RU	18.00	<0.0001
per01; elav>UAS-per24		2	100µg/ml RU489	321	59	per01 no RU / per01 +RU	1.72	ns
Control; elav>UAS-per10		1	etOH	413	52	Ctrl no RU / per01 no RU	17.31	<0.0001
Control; elav>UAS-per10		1	100µg/ml RU490	268	50	Ctrl no RU / Ctrl +RU	-3.85	<0.001
per01; elav>UAS-per10		1	etOH	424	61	Ctrl + RU/ per01 + RU	20.00	<0.0001
per01; elav>UAS-per10		1	100µg/ml RU491	299	60	per01 no RU / per01 +RU	-1.64	<0.05
Control; TIGS>UAS-per24	4C	1	etOH	350	52	Ctrl no RU / per01 no RU	13.46	<0.0001
Control; TIGS>UAS-per24	4C	1	100µg/ml RU486	373	52	Ctrl no RU / Ctrl +RU	0.00	ns
per01; TIGS>UAS-per24	4C	1	etOH	329	59	Ctrl + RU/ per01 + RU	0.00	ns
per01; TIGS>UAS-per24	4C	1	100µg/ml RU486	278	52	per01 no RU / per01 +RU	-11.86	<0.0001
Control; TIGS>UAS-per24		2	etOH	346	52	Ctrl no RU / per01 no RU	15.38	<0.0001
Control; TIGS>UAS-per24		2	100µg/ml RU486	355	50	Ctrl no RU / Ctrl +RU	-3.85	<0.01
per01; TIGS>UAS-per24		2	etOH	319	60	Ctrl + RU/ per01 + RU	4.00	<0.01
per01; TIGS>UAS-per24		2	100µg/ml RU486	265	52	per01 no RU / per01 +RU	-13.33	<0.0001
Control; TIGS>UAS-per10		1	etOH	319	48	Ctrl no RU / per01 no RU	22.92	<0.0001
Control; TIGS>UAS-per10		1	100µg/ml RU486	334	50	Ctrl no RU / Ctrl +RU	4.17	<0.001
per01; TIGS>UAS-per10		1	etOH	294	59	Ctrl + RU/ per01 + RU	6.00	<0.001
per01; TIGS>UAS-per10		1	100µg/ml RU486	258	53	per01 no RU / per01 +RU	-10.17	<0.0001
Control, NP3084>	4D	1	-	263	56	Ctrl, NP / per01, NP	17.86	<0.0001
Control, NP3084>UAS-per24	4D	1	-	378	54	Ctrl, NP/ Ctrl, NP>per	-3.57	<0.01
per01, NP3084>	4D	1	-	250	66	ctrl, NP>per/ per01,NP>per	0.00	ns
per01, NP3084>UAS-per24	4D	1	-	410	54	per01, NP / per01, NP>per	-18.18	<0.0001
Control, NP3084>		2	-	212	54	Ctrl, NP / per01, NP	20.37	<0.0001
Control, NP3084>UAS-per24		2	-	242	56	Ctrl, NP/ Ctrl, NP>per	3.70	<0.01
per01, NP3084>		2	-	229	65	ctrl, NP>per/ per01,NP>per	-10.71	<0.0001
per01, NP3084>UAS-per24		2	-	197	50	per01, NP / per01, NP>per	-23.08	<0.0001
Control, Esg>	4E	1	-	313	59	Ctrl, Esg / per01, Esg	16.95	<0.0001
Control, esg>UAS-per24	4E	1	-	394	63	Ctrl, Esg/ Ctrl, Esg>per	6.78	<0.01
per01, Esg>	4E	1	-	577	69	ctrl, Esg>per/ per01,Esg>per	-3.17	<0.01
per01, esg>UAS-per24	4E	1	-	260	61	per01, Esg / per01, Esg>per	-11.59	<0.0001
Control, Esg>		2	-	142	64	Ctrl, Esg / per01, Esg	12.50	<0.0001
Control, esg>UAS-per24		2	-	152	66	Ctrl, Esg/ Ctrl, Esg>per	3.13	<0.01
per01, Esg>		2	-	239	72	ctrl, Esg>per/ per01,Esg>per	-4.55	<0.001
per01, esg>UAS-per24		2	-	155	63	per01, Esg / per01, Esg>per	-12.50	<0.0001

Figure 4 continued

Genotype	Figure	rep	Drug/Diet	n	Median Lifespan	comparison	% change	log-rank p-value
Control; 5961>UAS-per24	4E	1	etOH	313	52	Ctrl no RU / per01 no RU	19.23	<0.0001
Control; 5961>UAS-per24	4E	1	100µg/ml RU486	358	54	Ctrl no RU / Ctrl +RU	3.85	<0.01
per01; 5961>UAS-per24	4E	1	etOH	532	62	Ctrl + RU/ per01 + RU	0.00	ns
per01; 5961>UAS-per24	4E	1	100µg/ml RU486	355	54	per01 no RU / per01 +RU	-12.90	<0.0001
Control; 5961>UAS-per24		2	etOH	213	51	Ctrl no RU / per01 no RU	17.65	<0.0001
Control; 5961>UAS-per24		2	100µg/ml RU486	311	51	Ctrl no RU / Ctrl +RU	0.00	ns
per01; 5961>UAS-per24		2	etOH	223	60	Ctrl + RU/ per01 + RU	1.96	ns
per01; 5961>UAS-per24		2	100µg/ml RU486	196	52	per01 no RU / per01 +RU	-13.33	<0.0001
Ctrl, daGS>UAS-CAS9, UAS-pergRNA	4F	1	etOH	187	50	Ctrl no RU / per01 no RU	18.00	<0.0001
Ctrl, daGS>UAS-CAS9, UAS-pergRNA	4F	1	100µg/ml RU486	251	58	Ctrl no RU / Ctrl +RU	16.00	<0.0001
per01, daGS>UAS-CAS9, UAS-pergRNA	4F	1	etOH	324	59	Ctrl + RU/ per01 + RU	0.00	ns
per01, daGS>UAS-CAS9, UAS-pergRNA	4F	1	100µg/ml RU486	269	58	per01 no RU / per01 +RU	-1.69	<0.01
Ctrl, daGS>UAS-CAS9, UAS-pergRNA		2	etOH	187	51	Ctrl no RU / per01 no RU	17.65	<0.0001
Ctrl, daGS>UAS-CAS9, UAS-pergRNA		2	100µg/ml RU486	265	58	Ctrl no RU / Ctrl +RU	13.73	<0.0001
per01, daGS>UAS-CAS9, UAS-pergRNA		2	etOH	269	60	Ctrl + RU/ per01 + RU	0.00	ns
per01, daGS>UAS-CAS9, UAS-pergRNA		2	100µg/ml RU486	285	58	per01 no RU / per01 +RU	-3.33	<0.01
TIGS>UAS-CAS9, UAS-pergRNA	4G	1	etOH	190	46			
TIGS>UAS-CAS9, UAS-pergRNA	4G	1	5µg/ml RU Dev	437	54	no RU vs Dev + RU	17.39	<0.0001
TIGS>UAS-CAS9, UAS-pergRNA	4G	1	100µg/ml RU486	233	58	no RU vs Adult + RU	26.09	<0.0001
5961>UAS-CAS9, UAS-pergRNA	4H	1	etOH	126	48			
5961>UAS-CAS9, UAS-pergRNA	4H	1	5µg/ml RU Dev	142	60	no RU vs Dev + RU	25.00	<0.0001
5961>UAS-CAS9, UAS-pergRNA	4H	1	100µg/ml RU486	141	60	no RU vs Adult + RU	25.00	<0.0001

Figure 5

Genotype	Figure	rep	Drug/Diet	n	Median Lifespan	comparison	% change	log-rank p-value
Control; TIGS>UAS-Ucp4C-RNAi	5D	1	etOH	280	43	Ctrl no RU / per01 no RU	27.91	<0.0001
Control; TIGS>UAS-Ucp4C-RNAi	5D	1	100µg/ml RU486	216	37	Ctrl no RU / Ctrl +RU	-13.95	<0.001
per01; TIGS>UAS-Ucp4C-RNAi	5D	1	etOH	293	55	Ctrl + RU/ per01 + RU	0.00	ns
per01; TIGS>UAS-Ucp4C-RNAi	5D	1	100µg/ml RU486	259	37	per01 no RU / per01 +RU	-32.73	<0.0001
Control; TIGS>UAS-Ucp4C-RNAi		2	etOH	193	47	Ctrl no RU / per01 no RU	31.91	<0.0001
Control; TIGS>UAS-Ucp4C-RNAi		2	100µg/ml RU486	112	40	Ctrl no RU / Ctrl +RU	-14.89	<0.001
per01; TIGS>UAS-Ucp4C-RNAi		2	etOH	105	62	Ctrl + RU/ per01 + RU	2.50	ns
per01; TIGS>UAS-Ucp4C-RNAi		2	100µg/ml RU486	119	41	per01 no RU / per01 +RU	-33.87	<0.0001
Control; 5961>UAS-Ucp4C-RNAi	5E	1	etOH	153	55	Ctrl no RU / per01 no RU	14.55	<0.0001
Control; 5961>UAS-Ucp4C-RNAi	5E	1	100µg/ml RU486	117	53	Ctrl no RU / Ctrl +RU	-3.64	<0.001
per01; 5961>UAS-Ucp4C-RNAi	5E	1	etOH	234	63	Ctrl + RU/ per01 + RU	0.00	ns
per01; 5961>UAS-Ucp4C-RNAi	5E	1	100µg/ml RU486	123	53	per01 no RU / per01 +RU	-15.87	<0.0001
Control; 5961>UAS-Ucp4C-RNAi		2	etOH	188	53	Ctrl no RU / per01 no RU	18.87	<0.0001
Control; 5961>UAS-Ucp4C-RNAi		2	100µg/ml RU486	167	51	Ctrl no RU / Ctrl +RU	-3.77	<0.001
per01; 5961>UAS-Ucp4C-RNAi		2	etOH	201	63	Ctrl + RU/ per01 + RU	0.00	ns
per01; 5961>UAS-Ucp4C-RNAi		2	100µg/ml RU486	126	51	per01 no RU / per01 +RU	-19.05	<0.0001
NP3084>+	5F	1	-	364	54			
UAS-Ucp4C>+	5F	1	-	315	52	NP>+ vs. NP>UCP4c	14.81	<0.0001
NP3084>UAS-Ucp4C	5F	1	-	287	62	UAS>+ vs. NP>UCP4c	19.23	<0.0001
NP3084>+		2	-	256	56			
UAS-Ucp4C>+		2	-	436	54	NP>+ vs. NP>UCP4c	10.71	<0.0001
NP3084>UAS-Ucp4C		2	-	291	62	UAS>+ vs. NP>UCP4c	14.81	<0.0001
NP3084>+		3	-	261	50			
UAS-Ucp4C>+		3	-	283	54	NP>+ vs. NP>UCP4c	24.00	<0.0001
NP3084>UAS-Ucp4C		3	-	274	62	UAS>+ vs. NP>UCP4c	14.81	<0.0001
Esg>+	5G	1	-	107	57			
UAS-Ucp4C>+	5G	1	-	131	59	Esg>+ vs. Esg>UCP4c	31.58	<0.0001
Esg>UAS-Ucp4C	5G	1	-	174	75	UAS>+ vs. Esg>UCP4c	27.12	<0.0001
Esg>+		2	-	122	58			
UAS-Ucp4C>+		2	-	145	56	Esg>+ vs. Esg>UCP4c	20.69	<0.0001
Esg>UAS-Ucp4C		2	-	118	70	UAS>+ vs. Esg>UCP4c	25.00	<0.0001
5961>UAS-Ucp4C	5H	1	etOH	140	51			
5961>UAS-Ucp4C	5H	1	100µg/ml RU487	254	61	no RU vs. + RU	19.61	<0.0001
5961>UAS-Ucp4C		2	etOH	212	52			
5961>UAS-Ucp4C		2	100µg/ml RU488	234	60	no RU vs. + RU	15.38	<0.0001
5961>UAS-Ucp4C		3	etOH	115	50			
5961>UAS-Ucp4C		3	100µg/ml RU489	101	59	no RU vs. + RU	18.00	<0.0001

Figure 7

Genotype	Figure	rep	Drug/Diet	n	Median Lifespan	comparison	% change	log-rank p-value
W1118 Canton S	7A	1	EtOH	243	51			
W1118 Canton S	7A	1	50µM DNP (all life)	261	60	Vehicle vs. DNP all life	17.65	<0.0001
W1118 Canton S	7B	1	EtOH	201	51			
W1118 Canton S	7B	1	50µM DNP Adult	199	61	Vehicle vs. DNP Adult	19.61	<0.0001
W1118 Canton S		2	EtOH	124	50			
W1118 Canton S		2	50µM DNP Adult	119	58	Vehicle vs. DNP Adult	16.00	<0.0001
W1118 Canton S	7C/S6C	-	EtOH	201	51	etOH Vs.		
W1118 Canton S	7C/S6C	-	10µM DNP Adult	207	56	10µM DNP Adult	9.80	<0.0001
W1118 Canton S	7C/S6C	-	50µM DNP Adult	199	61	50µM DNP Adult	19.61	<0.0001
W1118 Canton S	7C/S6C	-	100µM DNP Adult	198	49	100µM DNP Adult	-3.92	<0.01
esgts>UAS-Notch-RNAi	7H	1	EtOH	116	25			
esgts>UAS-Notch-RNAi	7H	1	50µM DNP Adult	88	44	Vehicle vs. DNP Adult	76.00	<0.0001
esgts>UAS-Notch-RNAi		2	EtOH	84	32			
esgts>UAS-Notch-RNAi		2	50µM DNP Adult	71	45	Vehicle vs. DNP Adult	40.63	<0.0001

Supplementary Figure 1

Genotype	Figure	rep	Drug/Diet	n	Median Lifespan	comparison	% change	log-rank p-value
Control; ubi-GAL4>		1	-	196	54	Ctrl ubi / per01 ubi	14.81	<0.0001
Control; ubi-GAL4>UAS-per24		1	-	190	52	Ctrl ubi / Ctrl ubi>per	-3.70	<0.01
per01; ubi-GAL4>		1	-	221	62	Ctrl ubi>per / per01 ubi>per	1.92	ns
per01; ubi-GAL4>UAS-per24		1	-	173	53	per01 ubi / per01 ubi>per	-14.52	<0.0001
Control; ubi-GAL4>	S1A	2	-	430	50	Ctrl ubi / per01 ubi	20.00	<0.0001
Control; ubi-GAL4>UAS-per24	S1A	2	-	181	52	Ctrl ubi / Ctrl ubi>per	4.00	ns
per01; ubi-GAL4>	S1A	2	-	535	60	Ctrl ubi>per / per01 ubi>per	-3.85	ns
per01; ubi-GAL4>UAS-per24	S1A	2	-	182	50	per01 ubi / per01 ubi>per	-16.67	<0.0001
Control; ubi-GAL4>		1	-	170	52	Ctrl ubi / per01 ubi	21.15	<0.0001
Control; ubi-GAL4>UAS-per10		1	-	203	52	Ctrl ubi / Ctrl ubi>per	0.00	ns
per01; ubi-GAL4>		1	-	227	63	Ctrl ubi>per / per01 ubi>per	3.85	0.04398
per01; ubi-GAL4>UAS-per10		1	-	170	54	per01 ubi / per01 ubi>per	-14.29	<0.0001
Control; ubi-GAL4>	S1A	2	-	209	52	Ctrl ubi / per01 ubi	17.31	<0.0001
Control; ubi-GAL4>UAS-per10	S1A	2	-	222	52	Ctrl ubi / Ctrl ubi>per	0.00	ns
per01; ubi-GAL4>	S1A	2	-	159	61	Ctrl ubi>per / per01 ubi>per	1.92	ns
per01; ubi-GAL4>UAS-per10	S1A	2	-	329	53	per01 ubi / per01 ubi>per	-13.11	<0.0001
Control; tim-GAL4>	S1B	1	-	158	50	Ctrl Tim / per01 Tim	24.00	<0.0001
Control; tim>UAS-per10	S1B	1	-	129	52	Ctrl Tim / Ctrl Tim>per10	4.00	ns
Control; tim>UAS-per24	S1B	1	-	103	46	Ctrl Tim / Ctrl Tim>per24	-7.69	<0.01
per01; tim-GAL4>	S1B	1	-	237	62	per01 Tim ctrl / per01 Tim>per10	-25.81	<0.0001
per01; tim-GAL4>UAS-per10	S1B	1	-	130	46	per01 Tim ctrl / per01 Tim>per24	-25.81	<0.0001
per01; tim-GAL4>UAS-per24	S1B	1	-	144	46	Ctrl Tim>per24 / per01 TIM>per24	0.00	ns
Control; daGS>		1	etOH	275	54	Ctrl no RU / per01 no RU	18.52	<0.0001
Control; daGS>		1	100µg/ml RU486	254	52	Ctrl no RU / +RU	-3.70	0.02983
per01; daGS>		2	etOH	291	64	Ctrl +RU / per01 +RU	23.08	<0.0001
per01; daGS>		2	100µg/ml RU486	311	64	per01 no RU / per01 +RU	0.00	ns
Control; daGS>	S1D	1	etOH	193	53	Ctrl no RU / per01 no RU	20.75	<0.0001
Control; daGS>	S1D	1	100µg/ml RU486	201	52	no RU / +RU	-1.89	ns
per01; daGS>	S1D	2	etOH	176	64	Ctrl + RU/ per01 + RU	19.23	<0.0001
per01; daGS>	S1D	2	100µg/ml RU486	182	62	per01 no RU / per01 +RU	-3.13	ns

Supplementary Figure 3

Genotype	Figure	rep	Drug/Diet	n	Median Lifespan	comparison	% change	log-rank p-value
Control; daGS>UAS-Ucp4A-RNAi	S3E	1	etOH	238	51	Ctrl no RU / per01 no RU	19.61	<0.0001
Control; daGS>UAS-Ucp4A-RNAi	S3E	1	100µg/ml RU486	415	51	Ctrl no RU / +RU	0.00	ns
per01; daGS>UAS-Ucp4A-RNAi	S3E	1	etOH	320	61	Ctrl +RU / per01 +RU	15.69	<0.0001
per01; daGS>UAS-Ucp4A-RNAi	S3E	1	100µg/ml RU486	370	59	per01 no RU / per01 +RU	-3.28	<0.01
Control; daGS>UAS-Ucp4A-RNAi		2	etOH	192	50	Ctrl no RU / per01 no RU	18.00	<0.0001
Control; daGS>UAS-Ucp4A-RNAi		2	100µg/ml RU486	189	49	no RU / +RU	-2.00	ns
per01; daGS>UAS-Ucp4A-RNAi		2	etOH	246	59	Ctrl + RU/ per01 + RU	20.41	<0.0001
per01; daGS>UAS-Ucp4A-RNAi		2	100µg/ml RU486	221	59	per01 no RU / per01 +RU	0.00	ns
Control; daGS>UAS-Ucp4B-RNAi	S3F	1	etOH	294	55	Ctrl no RU / per01 no RU	18.18	<0.0001
Control; daGS>UAS-Ucp4B-RNAi	S3F	1	100µg/ml RU486	258	51	Ctrl no RU / +RU	-7.27	<0.001
per01; daGS>UAS-Ucp4B-RNAi	S3F	1	etOH	231	65	Ctrl +RU / per01 +RU	-3.92	ns
per01; daGS>UAS-Ucp4B-RNAi	S3F	1	100µg/ml RU486	264	49	per01 no RU / per01 +RU	-24.62	<0.0001
Control; daGS>UAS-Ucp4B-RNAi		2	etOH	119	52	Ctrl no RU / per01 no RU	21.15	<0.0001
Control; daGS>UAS-Ucp4B-RNAi		2	100µg/ml RU486	213	50	no RU / +RU	-3.85	<0.001
per01; daGS>UAS-Ucp4B-RNAi		2	etOH	175	63	Ctrl + RU/ per01 + RU	0.00	ns
per01; daGS>UAS-Ucp4B-RNAi		2	100µg/ml RU486	154	50	per01 no RU / per01 +RU	-20.63	<0.0001
Control; daGS>UAS-Ucp4C-RNAi	S3G	1	etOH	262	57	Ctrl no RU / per01 no RU	14.04	<0.0001
Control; daGS>UAS-Ucp4C-RNAi	S3G	1	100µg/ml RU486	349	55	Ctrl no RU / +RU	-3.51	<0.01
per01; daGS>UAS-Ucp4C-RNAi	S3G	1	etOH	479	65	Ctrl +RU / per01 +RU	-3.64	<0.001
per01; daGS>UAS-Ucp4C-RNAi	S3G	1	100µg/ml RU486	423	53	per01 no RU / per01 +RU	-18.46	<0.0001
Control; daGS>UAS-Ucp4C-RNAi		2	etOH	212	55	Ctrl no RU / per01 no RU	20.00	<0.0001
Control; daGS>UAS-Ucp4C-RNAi		2	100µg/ml RU486	199	54	no RU / +RU	-1.82	<0.01
per01; daGS>UAS-Ucp4C-RNAi		2	etOH	189	66	Ctrl + RU/ per01 + RU	0.00	ns
per01; daGS>UAS-Ucp4C-RNAi		2	100µg/ml RU486	193	54	per01 no RU / per01 +RU	-18.18	<0.0001

Supplementary Figure 4

Genotype	Figure	rep	Drug/Diet	n	Median Lifespan	comparison	% change	log-rank p-value
daGS>+	S4C	1	etOH	279	54			
daGS>+	S4C	1	5µg/ml RU486	232	52	no RU / +RU	-3.70	<0.05
daGS>+		2	etOH	211	51			
daGS>+		2	5µg/ml RU486	214	51	no RU / +RU	0.00	ns

Supplementary Figure 5

Genotype	Figure	rep	Drug/Diet	n	Median Lifespan	comparison	% change	log-rank p-value
daGS>UAS-CAS9, UAS-CtrlgRNA	S5D	-	etOH	217	48			
daGS>UAS-CAS9, UAS-CtrlgRNA	S5D	-	5µg/ml RU Dev	194	40	no RU vs Dev + RU	-16.67	<0.0001
daGS>UAS-CAS9, UAS-CtrlgRNA	S5D	-	100µg/ml RU486	134	46	no RU vs Adult + RU	-4.17	<0.01
TIGS>UAS-CAS9, UAS-CtrlgRNA	S5E	-	etOH	157	46			
TIGS>UAS-CAS9, UAS-CtrlgRNA	S5E	-	5µg/ml RU Dev	140	48	no RU vs Dev + RU	4.35	<0.01
TIGS>UAS-CAS9, UAS-CtrlgRNA	S5E	-	100µg/ml RU486	114	45	no RU vs Adult + RU	-2.17	ns
5961>UAS-CAS9, UAS-CtrlgRNA	S5F	-	etOH	163	46			
5961>UAS-CAS9, UAS-CtrlgRNA	S5F	-	5µg/ml RU Dev	168	43	no RU vs Dev + RU	-6.52	<0.0001
5961>UAS-CAS9, UAS-CtrlgRNA	S5F	-	100µg/ml RU486	104	42	no RU vs Adult + RU	-8.70	<0.0001
Control; elav>	S5G	1	etOH	534	50	Ctrl no RU / per01 no RU	12.00	<0.0001
Control; elav>	S5G	1	100µg/ml RU486	500	50	Ctrl no RU / Ctrl +RU	0.00	ns
per01; elav>	S5G	1	etOH	788	56	Ctrl + RU/ per01 + RU	14.00	<0.0001
per01; elav>	S5G	1	100µg/ml RU487	663	57	per01 no RU / per01 +RU	1.79	<0.01
Control; elav>		2	etOH	554	46	Ctrl no RU / per01 no RU	21.74	<0.0001
Control; elav>		2	100µg/ml RU488	500	47	Ctrl no RU / Ctrl +RU	2.17	<0.01
per01; elav>		2	etOH	610	56	Ctrl + RU/ per01 + RU	21.28	<0.0001
per01; elav>		2	100µg/ml RU489	663	57	per01 no RU / per01 +RU	1.79	ns
Control; TIGS>	S5H	1	etOH	504	48	Ctrl no RU / per01 no RU	14.58	<0.0001
Control; TIGS>	S5H	1	100µg/ml RU486	554	46	Ctrl no RU / Ctrl +RU	-4.17	ns
per01; TIGS>	S5H	1	etOH	746	55	Ctrl + RU/ per01 + RU	19.57	<0.0001
per01; TIGS>	S5H	1	100µg/ml RU487	610	55	per01 no RU / per01 +RU	0.00	ns
Control; TIGS>		2	etOH	354	49	Ctrl no RU / per01 no RU	18.37	<0.0001
Control; TIGS>		2	100µg/ml RU488	312	51	Ctrl no RU / Ctrl +RU	4.08	<0.001
per01; TIGS>		2	etOH	310	58	Ctrl + RU/ per01 + RU	11.76	<0.0001
per01; TIGS>		2	100µg/ml RU489	333	57	per01 no RU / per01 +RU	-1.72	ns
Control; 5961>	S5I	1	etOH	136	56	Ctrl no RU / per01 no RU	10.71	<0.0001
Control; 5961>	S5I	1	100µg/ml RU486	126	56	Ctrl no RU / Ctrl +RU	0.00	ns
per01; 5961>	S5I	1	etOH	149	62	Ctrl + RU/ per01 + RU	10.71	<0.0001
per01; 5961>	S5I	1	100µg/ml RU487	202	62	per01 no RU / per01 +RU	0.00	ns
Control; 5961>		2	etOH	184	55	Ctrl no RU / per01 no RU	14.55	<0.0001
Control; 5961>		2	100µg/ml RU488	155	54	Ctrl no RU / Ctrl +RU	-1.82	ns
per01; 5961>		2	etOH	127	63	Ctrl + RU/ per01 + RU	14.81	<0.0001
per01; 5961>		2	100µg/ml RU489	146	62	per01 no RU / per01 +RU	-1.59	<0.05

Supplementary Figure 6

Genotype	Figure	rep	Drug/Diet	n	Median Lifespan	comparison	% change	log-rank p-value
elav-GS>UAS-Ucp4C	S6C	1	etOH	203	49			
elav-GS>UAS-Ucp4C	S6C	1	100µg/ml RU487	194	51	no RU vs. + RU	4.08	ns
elav-GS>UAS-Ucp4C		2	etOH	147	51			
elav-GS>UAS-Ucp4C		2	100µg/ml RU487	215	49	no RU vs. + RU	-3.92	ns
TIGS>UAS-Ucp4C	S6D	1	etOH	274	47			
TIGS>UAS-Ucp4C	S6D	1	100µg/ml RU488	267	53	no RU vs. + RU	12.77	<0.0001
TIGS>UAS-Ucp4C		2	etOH	238	49			
TIGS>UAS-Ucp4C		2	100µg/ml RU489	261	55	no RU vs. + RU	12.24	<0.0001

Supplementary Figure 7

Genotype	Figure	rep	Drug/Diet	n	Median Lifespan	comparison	% change	log-rank p-value
w1118,CS control		1	3% YE + Vehicle	375	54			
per01,w1118;CS;CS		1	3% YE + Vehicle	329	62	ctrl / per01	14.81	<0.0001
w1118,CS control	S7C	1	3% YE + Antibiotics	174	52			
per01,w1118;CS;CS	S7C	1	3% YE + Antibiotics	202	62	ctrl / per01	15.89	<0.0001
w1118,CS control		2	3% YE + Antibiotics	338	57			
per01,w1118;CS;CS		2	3% YE + Antibiotics	396	66	ctrl / per01	15.79	<0.0001

Supplementary Figure 8

Genotype	Figure	rep	Drug/Diet	n	Median Lifespan	comparison	% change	log-rank p-value
W1118 Canton S	S8A/D	-	EtOH	211	52	etOH Vs.		
W1118 Canton S	S8A/D	-	0.1 mM BHT Adult	158	58	0.1mM BHT Adult	11.54	<0.0001
W1118 Canton S	S8D	-	0.25 mM BHT Adult	178	54	0.25mM BHT Adult	3.85	<0.01
W1118 Canton S	S6D	-	1 mM BHT Adult	232	41	1mM BHT Adult	-21.15	<0.0001
per01; W1118 Canton S	S8D	-	EtOH	339	66	etOH Vs.		
per01; W1118 Canton S	S8D	-	0.1 mM BHT Adult	228	63	0.1mM BHT Adult	-4.55	<0.0001
per01; W1118 Canton S	S8D	-	0.25 mM BHT Adult	211	58	0.25mM BHT Adult	-12.12	<0.0001
per01; W1118 Canton S	S8D	-	1 mM BHT Adult	268	54	1mM BHT Adult	-18.18	<0.01
W1118 Canton S	S8C/7C	-	EtOH	201	51	etOH Vs.		
W1118 Canton S	S8C/7C	-	0.01 mM DNP Adult	207	58	10µM DNP Adult	13.73	<0.0001
W1118 Canton S	S8C/7C	-	0.05 mM DNP Adult	199	61	50µM DNP Adult	19.61	<0.0001
W1118 Canton S	S8C/7C	-	0.1 mM DNP Adult	198	49	100µM DNP Adult	-3.92	<0.01
per01; W1118 Canton S	S8C	-	EtOH	267	63	etOH Vs.		
per01; W1118 Canton S	S8C	-	0.01 mM DNP Adult	151	58	10µM DNP Adult	-7.94	<0.01
per01; W1118 Canton S	S8C	-	0.05 mM DNP Adult	206	58	50µM DNP Adult	-7.94	<0.001
per01; W1118 Canton S	S8C	-	0.1 mM DNP Adult	157	56	100µM DNP Adult	-11.11	<0.0001
