# Supplementary material for: Circadian regulation of mitochondrial uncoupling and lifespan
Source: Nat Commun. 2020 Apr 21;11:1927. doi: 10.1038/s41467-020-15617-x (PMC7174288; doi:10.1038/s41467-020-15617-x)
Supplement: Supplementary file 2 — Reporting Summary [file 41467_2020_15617_MOESM2_ESM.pdf]

nature research

Corresponding author(s): Michele Shirasu-Hiza

Last updated by author(s): Mar 10, 2020

Reporting Summary

Nature Research wishes to improve the reproducibility of the work that we publish. This form provides structure for consistency and transparency in reporting. For further information on Nature Research policies, see Authors & Referees and the Editorial Policy Checklist.

Statistics

For all statistical analyses, confirm that the following items are present in the figure legend, table legend, main text, or Methods section.

n/a	Confirmed	
☐	☒	The exact sample size (*n*) for each experimental group/condition, given as a discrete number and unit of measurement
☐	☒	A statement on whether measurements were taken from distinct samples or whether the same sample was measured repeatedly
☐	☒	The statistical test(s) used AND whether they are one- or two-sided<br>*Only common tests should be described solely by name; describe more complex techniques in the Methods section.*
☐	☒	A description of all covariates tested
☐	☒	A description of any assumptions or corrections, such as tests of normality and adjustment for multiple comparisons
☐	☒	A full description of the statistical parameters including central tendency (e.g. means) or other basic estimates (e.g. regression coefficient) AND variation (e.g. standard deviation) or associated estimates of uncertainty (e.g. confidence intervals)
☐	☒	For null hypothesis testing, the test statistic (e.g. *F*, *t*, *r*) with confidence intervals, effect sizes, degrees of freedom and *P* value noted<br>*Give P values as exact values whenever suitable.*
☒	☐	For Bayesian analysis, information on the choice of priors and Markov chain Monte Carlo settings
☒	☐	For hierarchical and complex designs, identification of the appropriate level for tests and full reporting of outcomes
☒	☐	Estimates of effect sizes (e.g. Cohen's *d*, Pearson's *r*), indicating how they were calculated

Our web collection on statistics for biologists contains articles on many of the points above.

Software and code

Policy information about availability of computer code

Data collection	Microsoft Excel was used to input and organize raw data. DatLab was used for the collection of oxygen consumption measurements from mitochondria (Oroboros O2k). BioRad CFX real time software was used for the collection of QPCR data. Zen Black software from Zeiss was used for the collection of fluorescent microscopy images.
Data analysis	GraphPad Prism version 7 was used for all statistical analysis, with the exception of locomotor activity analysis which was performed in ClockLab, http://www.actimetrics.com/products/clocklab/clocklab-analysis-version-6/

For manuscripts utilizing custom algorithms or software that are central to the research but not yet described in published literature, software must be made available to editors/reviewers. We strongly encourage code deposition in a community repository (e.g. GitHub). See the Nature Research guidelines for submitting code & software for further information.

Data

Policy information about availability of data

All manuscripts must include a data availability statement. This statement should provide the following information, where applicable:

- Accession codes, unique identifiers, or web links for publicly available datasets
- A list of figures that have associated raw data
- A description of any restrictions on data availability

All raw data that support the findings in this manuscript are available from the corresponding authors upon reasonable request.

Field-specific reporting

Please select the one below that is the best fit for your research. If you are not sure, read the appropriate sections before making your selection.

☒ Life sciences ☐ Behavioural & social sciences ☐ Ecological, evolutionary & environmental sciences

For a reference copy of the document with all sections, see nature.com/documents/nr-reporting-summary-flat.pdf

Life sciences study design

All studies must disclose on these points even when the disclosure is negative.

Sample size

For lifespan studies, we aimed to have 250 animals per condition, as in standard power analyses this gave us sufficient power to detect 5% changes in longevity with low type I and type II error. For biochemical analysis, we used independent biological replicates (3-10) of cohorts of at least 10 animals for all experiments. This allowed us statistical power to show only larger changes between conditions (e.g. 25% change) with low error, but proved sufficient in many cases.

Data exclusions

Only escaped Drosophila or alive immobilized (stuck in media) Drosophila were censored. Log-rank analysis with or without censoring made no significant difference in statistical conclusions as the number of animals censored was minimal or none for most experiments.

Replication

Lifespan experiments were reproduced at least twice whenever possible, and in some instances experiments were conducted four times to similar results. Biochemical analyses were conducted on independent biological replicates of at least three cohorts of animals per condition. Whenever possible these experiments were repeated with similar numbers multiple times (usually 3 independent experiments). More detailed information is found in the manuscript pertaining to each experimental assay including the number of repetitions.

Randomization

Allocation of preassigned animals to specific groups was random within genotype, and treatment.

Blinding

Blinding was performed for many of the initial lifespan experiments. Animal cohorts of specific genotype were assigned numbers and handed off to researchers (not involved in the number assignment) to score for death on individual days. Later experiments and biochemical experiments were not blinded due to the number of genotypes, assays performed, and time involved.

Reporting for specific materials, systems and methods

We require information from authors about some types of materials, experimental systems and methods used in many studies. Here, indicate whether each material, system or method listed is relevant to your study. If you are not sure if a list item applies to your research, read the appropriate section before selecting a response.

Materials & experimental systems

n/a	Involved in the study	
☐	☒	Antibodies
☒	☐	Eukaryotic cell lines
☒	☐	Palaeontology
☐	☒	Animals and other organisms
☒	☐	Human research participants
☒	☐	Clinical data

Methods

n/a	Involved in the study	
☒	☐	ChIP-seq
☒	☐	Flow cytometry
☒	☐	MRI-based neuroimaging

Antibodies

Antibodies used

AMPK phospho-T184 at 1:1000 (Cell Signaling, 40H9-2535); anti-phospho-S6K T398 (Cell Signaling, 9209), anti-GFP (Cell Signaling, rabbit D5.1, mouse 4B10), anti-phospho AKT s505 (Cell Signaling , 4054), and pan AKT at 1:1000 (Cell Signaling, 4691); and horseradish peroxidase (HRP)-conjugated monoclonal mouse anti-actin antibody at 1:5000 (Sigma-Aldrich, A3854). anti-phospho Histone H3 (S10) (Cell Signaling, 4499) Rabbit antibodies were detected using HRP-conjugated anti-rabbit IgG antibodies at 1:2000 (Cell Signaling, 7074) and AlexaFluor-488 (Thermo Fisher, A-11034). Mouse antibodies were detected using HRP-conjugated anti-mouse IgG antibodies 1:2000 dilution (Cell Signaling, 7076)

Validation

Validation of all antibodies is available from manufacturer website.

Animals and other organisms

Policy information about studies involving animals; ARRIVE guidelines recommended for reporting animal research

Laboratory animals

cycle (cyc01) period (per01) and timeless (tim01) mutants outcrossed to the Canton-S (CS) background as controls were obtained from Jaga Giebultowicz. pBAC-Ucp4ce03988, UASDN-S6K (6911) were obtained from the Bloomington Stock Center. UAS-Ucp4a-RNAi, UAS-Ucp4b-RNAi, UAS-Ucp4c-RNAi were obtained from the Vienna Drosophila Resource Center. UAS-Ucp4c

was received from flyORF. UAS-GFP-Atg8 from Eric Baehrecke, Ubi-GAL4 from Marc Dionne, Esg-GAL4, NP3084-GAL4, Daughterless-Gene-Switch (DaGS), Elav-Gene-Switch (ELAV-GS) and TIGS-2 (TIGS-GAL4), 5961GSGAL4, DILP2-GAL4 and UAS-Reaper from David W. Walker, UAS-per10 and UAS-per24, and UAS-DN-Clock from Amita Sehgal, Esgts-GAL4, and UAS-Notch-RNAi were from Ben Ohlstein. All experiments with multiple transgenes used flies that have undergone 12 generations of out-crossing into a w1118 Canton-S (CS) control and/or per01, w1118 Canton-S (CS) mutant background.

Wild animals	No wild animals were used in this study
Field-collected samples	No field collected samples were used in this study
Ethics oversight	All guidelines for the proper treatment and disposal of recombinant Drosophila were used.

Note that full information on the approval of the study protocol must also be provided in the manuscript.
